# Honey bee sHSP are responsive to diverse proteostatic stresses and potentially promising biomarkers of honey bee stress

**DOI:** 10.1038/s41598-021-01547-1

**Published:** 2021-11-11

**Authors:** Samantha R. Shih, Dunay M. Bach, Nicole C. Rondeau, Jessica Sam, Natalie L. Lovinger, Allison J. Lopatkin, Jonathan W. Snow

**Affiliations:** Biology Department, Barnard College, New York, NY 10027 USA

**Keywords:** Chaperones, Protein aggregation, Entomology

## Abstract

The pollination services provided by the honey bee are critical in both natural and agricultural ecosystems. Honey bee colonies in the United States have suffered from an increased rate of die-off in recent years, stemming from a complex set of interacting stresses that remain poorly described. Defining specific common cellular processes and cellular stress responses impacted by multiple stressors represent a key step in understanding these synergies. Proteotoxic stresses negatively impact protein synthesis, folding, and degradation. Diverse proteotoxic stresses induce expression of genes encoding small heat shock proteins (sHSP) of the expanded *lethal (2) essential for life* (*l(*2*)efl*) gene family. In addition to upregulation by the Integrated Stress Response (ISR), the Heat Shock Response (HSR), and the Oxidative Stress Response (OSR), our data provide first evidence that sHSP genes are upregulated by the Unfolded Protein Response (UPR). As these genes appear to be part of a core stress response that could serve as a useful biomarker for cellular stress in honey bees, we designed and tested an RT-LAMP assay to detect increased *l(2)efl* gene expression in response to heat-stress. While this assay provides a powerful proof of principle, further work will be necessary to link changes in *sHSP* gene expression to colony-level outcomes, to adapt our preliminary assay into a Point of Care Testing (POCT) assay appropriate for use as a diagnostic tool for use in the field, and to couple assay results to management recommendations.

## Introduction

Honey bee pollination services are critical for the health of both agricultural and ecological systems^1^. Honey bee colonies in the United States have been lost at an increased rate in since the mid-2000s. Stressors including nutritional deficiencies due to loss of appropriate habitat, toxicity from pesticides, changes to normal living conditions brought about through industrial beekeeping practices, a host of environmental changes due to climate change, and parasitism by arthropod pests and pathogenic microbes, likely work in concert to cause tissue pathology, disease and mortality^2^. While studies on the physiological stress responses in the honey bee have provided significant insight^3^, we are only beginning to understand honey bee cellular stress responses, especially at the molecular level. Attempts to more completely define common cellular processes and cell stress pathways have been undertaken to better understand how stresses might work together to cause honey bee disease.

Proteostasis, which is defined as the homeostasis of protein synthesis, folding, function, and degradation, represents one such process^4^. This process can be disrupted by a number of normal and pathologic conditions, ultimately leading to an accumulation of misfolded proteins in the cell^4^. The pathways of the proteostatic network maintain protein homeostasis in individual cells. These responses include the Unfolded Protein Response of the endoplasmic reticulum (UPR), which responds to proteostatic perturbation in this compartment^5^, the Integrated Stress Response (ISR), which responds to amino acid deprivation or ribosome dysfunction, and the Heat Shock Response (HSR), responding to disruption of proteostasis in the cytoplasm.

We recently used an unbiased approach to identify novel genes altered in expression during induction of these proteostatic network responses in the honey bee using transcriptome profiling (RNASeq). We used tunicamycin, which disrupts *N*-glycosylation in the ER to induce the UPR^6^. We also used thermal stress which causes protein denaturation and found that this induces a tissue regeneration program^7^. Finally, we used halofuginone, which inhibits the prolyl-tRNA synthetase to induce ISR, and observed increased expression of ribosome biogenesis genes^8^. We hypothesized that we could then utilize this information to better understand how these stresses interact to impact honey bee health and to identify candidate biomarkers of stress in honey bees.

## Materials and methods

### Honey bee tissue collection

Honey bees represent a typical mix of *Apis mellifera* subspecies found in North America. They were collected from the landing board of outbred colonies in New York, New York at different times during the months of April–October. Only visibly healthy bees were selected and all source colonies were routinely inspected for symptoms of common bacterial, fungal, and viral diseases of honey bees. All bees used in a given experiment were taken from the same colony. Following cold anesthesia, the following tissues were dissected for gene expression analysis: head tissue (predominantly brain, sensory organ tissue, and hypopharyngeal glands), midgut, thorax tissue (predominantly flight muscle), and abdominal wall tissue (predominantly fat body). Dissected tissues were immersed in RNAlater (Invitrogen, San Diego, CA) for storage prior to RNA extraction.

### Temperature and chemical treatments

Honey bees collected as above were maintained in 177.4 mL (6 oz.) Square-bottomed Drosophila Stock Bottles (VWR) plugged foam tube plugs (Jaece Industries, North Tonawanda, NY) modified to allow for feeding via microcentrifuge tubes as previously described^9^. Caged bees were maintained in incubators at 35 °C (except for during heat shock as described below). PseudoQueen strips (Contech, Victoria, British Columbia, Canada) were placed in the incubators as a source of Queen Mandibular Pheromone (QMP). Heat shocked bees were maintained for 4 h in cages at 45 °C. Bees were fed 33% sucrose via a modified 1.5 ml microcentrifuge tube, with 0.01–2.5 mM paraquat, 0.0001–0.001% NaArsenite, 24 µM tunicamycin, 200 µM halofuginone, or 0.5 mg/ml cycloheximide for 24 h. For controls, bees were fed sucrose solution alone for paraquat, NaArsenite or cycloheximide. As the solvent for tunicamycin and halofuginone is DMSO, equivalent amounts of DMSO were added to the sucrose solution of the control group experiments using those compounds.

### RNA isolation, reverse-transcription and quantitative PCR for gene expression analysis

RNA was prepared from bees from the described tissues as previously described^9^. Tissues were first manually crushed with a disposable pestle in Trizol Reagent (Invitrogen, San Diego, CA, USA) and RNA was then extracted as per the manufacturer’s instructions. RNA was then DNaseI treated by RQ1 RNase-Free DNase (Promega, Madison, WI, USA) and quantified. cDNA was synthesized using approximately 1 μg of RNA with the High-Capacity cDNA Reverse Transcription Kit with RNase Inhibitor (Applied Biosystems, Foster City, CA, USA). For quantitative PCR (qPCR) reactions to determine the expression levels of genes of interest, 1 μl of cDNA was used as a template in conjunction with PowerUP SYBR Green Master Mix (Applied Biosystems, Foster City, CA, USA) and appropriate primers. Reactions were run in a LightCycler 480 thermocycler (Basel, Switzerland). Primer sequences targeting transcripts of gene of interest developed for this study are in Supplementary Table 1. Primer sequences for the reference gene *β-actin* were from^10^. The difference between the threshold cycle number for *β-actin* and that of the gene of interest was used to calculate the level of that gene relative to *β-actin* using the typical 2^(−ΔCT)^ method^11^. All qPCR data represents expression values from individual bees (sample sizes found in figure legends) and is displayed as mean ± SEM. For a given experiment, bees were collected at the same time from a single colony. Each experiment was performed in at least 3 independent trials done on different days. Independent trials sourced bees from different colonies.

### RNA-Seq

For RNA-Seq analysis, we used data sets generated previously^6–8^. Briefly, we performed transcriptome profiling (RNASeq) on midguts from bees: (1) fed sucrose solution containing 24 μM Tunicamycin or DMSO for 24 h^6^, (2) maintained at either 35 or 45 °C for 4 h^7^, or (3) treated with sucrose solution containing 200 µM halofuginone or DMSO for 24 h^8^. RNASeq analysis was performed on 3 midguts from each group individually. Libraries were prepared using the NEBNext Ultra RNA library preparation kit and then sequenced using the paired end 150 bp sequencing configuration on the Illumina HiSeq 4000 platform. After the sequence reads were trimmed, they were then mapped to the *Apis mellifera* reference genome then available on NCBI (Amel 4.5 version). After mapping and total gene hit counts calculation, the total gene hit counts table was used for downstream differential expression analysis using DESeq2. The original p-values are generated using the Wald test. The adjusted/corrected p-values are obtained using the Benjamini and Hochberg method. Genes with adjusted p-value < 0.05 and Absolute Log2Fold Change > 1 were called as significant differentially expressed genes for each comparison. The RNA sequence information in this study was previously submitted to the Gene Expression Omnibus database under the accession numbers GSE139368, GSE159083, and GSE165411 (https://www.ncbi.nlm.nih.gov/). Venn diagram analysis of gene lists from RNA-Seq data (described previously) was performed using the following webtool (http://bioinformatics.psb.ugent.be/beg/tools/venn-diagrams).

### Identification of genes encoding l(2)efl proteins in bee (Hymenoptera: Apoidea: Anthophila) genomes

For a complete list of bee l(2)efl proteins, we used the amino acid sequence of *Drosophila melanogaster* l(2)efl protein (NP_001261156.1) as the query sequence to search select bee genomes in NCBI for which non-redundant protein sequences are available (*Apis mellifera*, *Apis cerana*, *Megachile rotundata*, *Eufriesea mexicana*, *Bombus impatiens*, *Habropoda laboriosa*, *Dufourea novaeangliae*, *Nomia melanderi*, *Osmia lignaria*, *Melipona quadrifasciata*, *Frieseomelitta varia, Megalopta genalis*, and *Ceratina calcarata*). To generate our alignment, we used the amino acid sequences of the *Drosophila melanogaster* and bee l(2)efl proteins. The heat shock protein beta-8 of *Homo sapiens* (NP_055180.1) was used as the outgroup. Protein alignments were generated with MUSCLE 2^12^ using default parameters and inspected manually. A maximum-likelihood phylogenetic tree was inferred from the resulting alignment using the RaxML program version 8^13^ with the GAMMA model. Bootstrapping was conducted with 100 replicates. The resulting tree was visualized and annotated using ggtree in R. For bee species for which genome annotation was available, we generated graphical representations (not to scale) of the *l(2)efl* genes in the cluster as well as those outside the cluster (Supplementary Fig. 2). The presence of signal sequences was predicted using Signal 4.1P (http://www.cbs.dtu.dk/services/SignalP/).

### RT-LAMP assay

We used RNA extracted as above in conjunction with WarmStart Colorimetric RT-LAMP 2X Master Mix or WarmStart LAMP Kit (DNA & RNA) (New England Biolabs, Ipswich, MA) and LAMP primers designed using NEB LAMP Primer Design Tool. NEB LAMP Primer Design Tool (Supplementary Table 1). Reactions mixes and conditions were set up according to the manufacturer’s instructions. Real-time fluorescence detection of products was performed using an iCycler thermo-cycler (Biorad, Hercules, CA, USA). For real-time fluorescence results, mean ± SEM is shown and represents relative expression values of *l(2)efl 724367* of individual bees calculated using a 2^(−ΔCT)^ method where no reference gene is employed, but instead ΔCT is determined by subtracting individual sample values from the mean CT value of the 35 °C group. For colorimetric results, reactions were stopped at 30 min and color changes assessed visually. All RT-LAMP data was confirmed in at least 3 independent experiments.

### Statistical analysis

Gene expression data was log10 transformed and compared using unpaired t-tests with Welch’s correction when values fit normal distributions. We used Mann–Whitney U nonparametric tests when data did not fit normal distributions. We evaluated data normality using Shapiro–Wilk tests. Statistics details can be found in Supplementary Table 2. Survival analysis data was analyzed using Log-rank (Mantel-Cox) test.

## Results

### UPR, ISR, and HSR alter the expression of *l(2)efl* sHSP genes in honey bees

We used Venn diagram analysis to identify a set of differentially expressed genes found in the individual UPR, HSR, and ISR data sets^6–8^. We identified increased and decreased expression of genes involved in multiple processes, but a striking finding was that four genes encoding sHSP (*l(2)efl* family genes *410087*, *724367, 724274*, and *724449*) were induced in all datasets (Supplementary Table 3). As sHSP genes are involved in stabilizing early unfolded intermediates of proteins and preventing further unfolding and formation of insoluble aggregates (reviewed in Refs.^14–17^), these genes appeared to be ideally suited to address diverse proteotoxic stressors.

### Bees possess an expanded (l)efl gene family

We previously examined the honey bee genome and identified fourteen genes encoding proteins containing the alpha-crystallin domain characteristic of sHSP (Ref.^9^ and Supplementary Tables 3, 4). Ten of these proteins share close homology to sHSP proteins characterized in *D. melanogaster*. Of these, one is a *Heat-shock protein β1* (*Hspβ1*) homology while the other nine are most closely related to *lethal (2) essential for life* (*l(2)efl*)^18^. Examination of proteins from select insects representing other orders shows a range in the number of sHSP proteins of the *l(2)efl* family, from 1 in *D. melanogaster* to 12 in *Folsomia candida* (Supplementary Table 5). None of the honey bee *l(2)efl* proteins are predicted to contain a signal sequence (Supplementary Table 4).

Gene *410087* was originally annotated as being a four-exon gene encoding a protein with two alpha-crystallin domains. PCR-based methods failed to find evidence for this gene model, instead suggesting the existence of two genes, which we will call *410087a* and *410087b* (both of which are closest to LOC724488 in protein sequence). In addition, gene models from the annotation of genomes from other *Apis* species (*Apis dorsata*, *Apis florea*, and *A. cerana*) support the model that *410087* is in fact two genes. The proposed mRNA and protein sequences for *410087a* and *410087b* are included in Supplementary Fig. 1.

**Figure 1:**
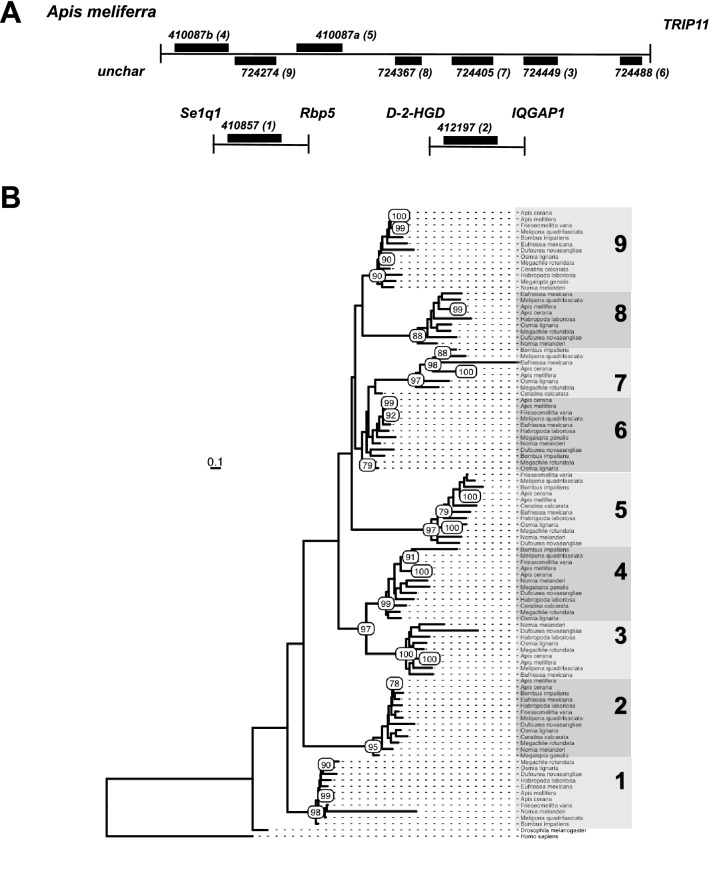
*l(2)efl* genes and proteins in bees. Schematic of the gene organization of *l(2)efl* genes in select bee species (both genes found in the cluster as well as genes found outside) (**A**). Phylogenetic tree of *D. melanogaster* l(2)efl proteins, bee l(2)efl proteins (see “Methods”), and *H. sapiens* alpha-crystallin B protein based on full amino acid sequences. Scale bar represents corresponds to the number of amino acid changes per site (**B**).

Seven of the genes encoding *l(2)efl* in the honey bee genome are located together in a cluster (Fig. 1A), while the other two are located at other sites in the genome. To explore conservation of this gene cluster in bees and to better understand their relationship to the single *l(2)efl* genes protein found in *D. melanogaster*, we used the amino acid sequence of the single *D. melanogaster l(2)efl* to search for *l(2)efl* encoding genes in select other bee genomes *A. cerana*, *M. rotundata*, *E. mexicana*, *B. impatiens*, *H. laboriosa*, *D. novaeangliae*, *N. melanderi*, *O. lignaria*, *M. quadrifasciata*,* M.*, and *C. calcarata*). These genomes represent both social and solitary bee species which are broadly distributed phylogenetically within the Clade Anthophila^19^. We found that all bees possessed between 4 and 9 genes encoding *l(2)efl* sHSP (Supplementary Table 6). For all bee species (except *C. calcarata and M. genali)* 6–7 clustered *l(2)efl* genes that were located in a single cluster between thyroid receptor-interacting protein 11 (LOC411348 in *A. mellifera*) and a gene encoding an uncharacterized protein (LOC100576174 in *A. mellifera*), while 1–2 *(2)efl* genes were located elsewhere in the genome.

We used the *l(2)efl* proteins from above as well as those from *F. varia* and *M. quadrifasciata* (bee species for which completely assembled and annotated genomes are not available) to construct a phylogenetic tree. The alpha-crystallin B chain protein of *Homo sapiens* (NP_001276736.1) was used as the outgroup. (Fig. 1B). As expected, we found that the bee *l(2)efl* proteins are grouped together on the tree and thus appear to share a common relative that expanded in number in bees.

### UPR and ISR both induce a subset of *l(2)efl* sHSP genes in honey bees

**Figure 2:**
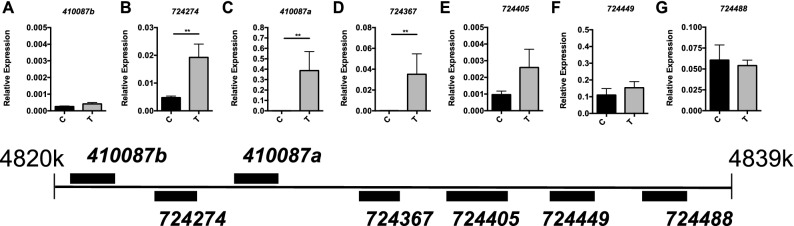
Select sHSP genes are induced during UPR induction. Transcript levels of the *l(2)efl* genes *410087b* (**A**), *724274* (**B**), *410087a* (**C**), *724367* (**D**)*, 724405* (**E**), *724449* (**F**), and *724488* (**G**) relative to *β-actin* in midgut tissue from adult bees captured at the landing board and fed sucrose solution containing tunicamycin (T, n = 8) or vehicle alone (C, n = 8) for 24 h. A schematic diagram of the colocalized *l(2)efl* genes in the honey bee genome is shown below for reference. Mean ± SEM is shown and represents expression values of the genes of interest calculated using the 2^(−ΔCT)^ method for individual bees. Statistical significance is noted as *p < 0.05, and **p < 0.01.

Examination of all *l(2)efl* genes in three RNAseq datasets showed that in addition to *724367*, *410087a,* and *724449,* the *l(2)efl* gene *724274* was induced above the cut-off (an adjusted p value of less than 0.05 and a Log2 Fold Change of greater than 1) in the UPR and HSR datasets and was very close to reaching the threshold in ISR dataset and the *724405* gene was induced above the cut-off in the ISR and HSR datasets and was very close to reaching the threshold in UPR dataset. Thus, 6 out of the 7 *l(2)efl* genes in the cluster were upregulated by at least 2 of the 3 stressors (even with the low sample number used for the original RNAseq). We then examined the expression of all of the cluster-localized *l(2)efl* genes by qPCR. We confirmed that expression of *724274*, *724367*, *410087a* were increased in the midguts of bees treated with tunicamycin relative to control bees (Fig. 2A–G). Statistics details can be found in Supplementary Table 2.

**Figure 3:**
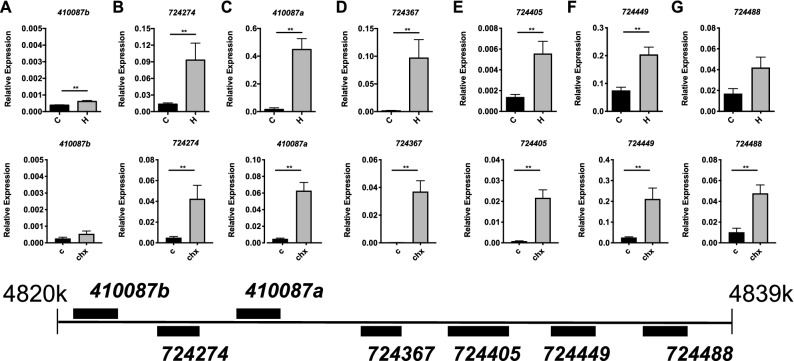
Select sHSP genes are induced during ISR induction. Transcript levels of the *l(2)efl* genes *410087b* (**A**), *724274* (**B**), *410087a* (**C**), *724367* (**D**)*, 724405* (**E**), *724449* (**F**), and *724488* (**G**) relative to *β-actin* in midgut tissue from adult bees captured at the landing board and fed sucrose solution containing halofuginone (H, n = 8) or vehicle alone (C, n = 8) for 24 h (top row). Transcript levels of these same genes relative to *β-actin* in midgut tissue from adult bees captured at the landing board and fed sucrose solution alone (C, n = 8) or with 0.5 mg/ml cyclohexamide for 48 h (chx, n = 8) (bottom row). A schematic diagram of the colocalized *l(2)efl* genes in the honey bee genome is shown below for reference. Mean ± SEM is shown and represents expression values of the genes of interest calculated using the 2^(−ΔCT)^ method for individual bees. Statistical significance is noted as *p < 0.05, and **p < 0.01.

We examined the relative expression of these *l(2)efl* genes after ISR activation and found that 5 out of 7 were upregulated relative to control bees after halofuginone treatment (Fig. 3A–G, top). *β-actin* levels were similar irrespective of tunicamycin or halofuginone treatment as assessed by Ct values (Supplementary Fig. 5). Other work in our lab has suggested that the cellular effects of halofuginone treatment may be due to a disruption in translation as opposed to an amino acid shortage^8^. To investigate whether a direct inhibition of translation leading to RSR activation could also induce *l(2)efl* sHSP, we used cycloheximide (CHX), which inhibits the translocation step in protein synthesis by binding to the ribosome, to block translational elongation. We observed that CHX treatment for 48 h induces expression of the *l(2)efl* genes *724488*, *724405*, *724367*, *724274*, *724449*, *410087a,* but not *410087b* in the midgut (Fig. 3A–G, bottom). Statistics details can be found in Supplementary Table 2.

### HSR induces all *l(2)efl* sHSP in honey bees

**Figure 4:**
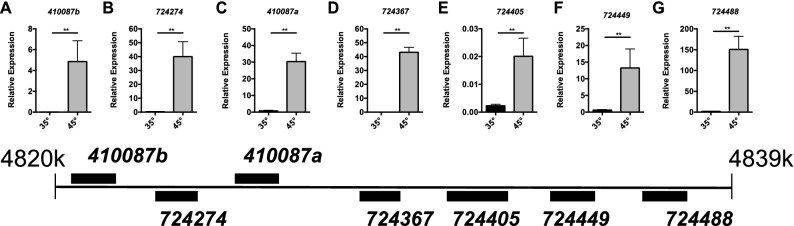
Select sHSP genes are induced during Heat-Shock. Transcript levels of the *l(2)efl* genes *410087b* (**A**), *724274* (**B**), *410087a* (**C**), *724367* (**D**)*, 724405* (**E**), *724449* (**F**), and *724488* (**G**) after heat shock relative to *β-actin* in midgut tissue from adult bees captured at the landing board and maintained for 4 h in cages at either 35° (n = 6) or 45 °C (n = 6). A schematic diagram of the colocalized *l(2)efl* genes in the honey bee genome is shown below for reference. Mean ± SEM is shown and represents expression values of the genes of interest calculated using the 2^(−ΔCT)^ method for individual bees. Statistical significance is noted as *p < 0.05, and **p < 0.01.

All of the *l(2)efl* homologs (except *410857*) located were induced by heat shock in our dataset (Supplementary Table 1). To confirm these results, we examined heat-shock dependent induction of genes encoding proteins that are homologs of existing sHSP (all *l(2)efl* homologs and *Hspβ1*) in midgut tissue. We observed increased levels of transcripts from the *l(2)efl* genes *724488*, *724405*, *724367*, *724274*, *724449*, as well as the *410087a* and *410087b* in the midguts of bees heat shocked at 45 °C for 4 h when compare to bees maintained at 35 °C for that period (Fig. 4). While orally administered treatments appear to reliably only affect the digestive tract, we can apply thermal stress to all tissues equally. Thus, we examined *shsp* expression in multiple tissues after heat shock. We also observed increased levels of transcripts from the *l(2)efl* genes *724488*, *724405*, *724367*, *724274*, *724449*, *410087a* and *410087b* after heat shock in other tissues, including the head, thorax, and abdominal wall tissue (Supplementary Fig. 3). Transcripts from the genes *l(2)efl* (410857) and *Heat shock protein beta-1 (Hspβ1)* were unchanged after heat shock (Supplementary Fig. 4). *β-actin* levels were similar irrespective of temperature as assessed by Ct values (Supplementary Fig. 5). Statistics details can be found in Supplementary Table 2.

### Oxidative stress induces a subset of *l(2)efl* sHSP in honey bees

**Figure 5:**
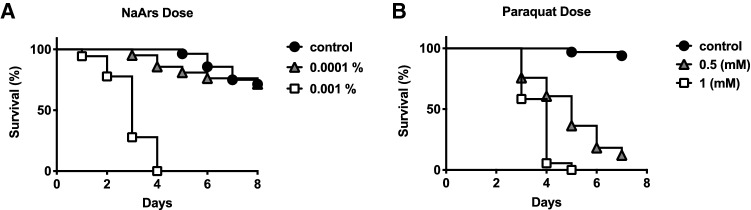
Oxidative stress mediated by NaArs or Paraquat ingestion reduces honey bee survival. (**A**) Survival of individual honey bees fed sugar solution containing 0 (n = 28), 0.001% (n = 18) or 0.0001% (n = 21) NaArs. Log-rank (Mantel-Cox) test showed that the curves are different (Chi square = 71.83, df = 2, < 0.0001). (**B**) Survival of individual honey bees fed sugar solution containing 0 (n = 33), 1 mM (n = 36), or 0.1 mM (n = 33) paraquat. Log-rank (Mantel-Cox) test showed that the curves are different (Chi square = 90.79, df = 2, < 0.0001).

Oxidative stress is another stressor that can impact proteostasis. We used sodium arsenite (NaArs) and paraquat to model oxidative stress-induced responses in the digestive tract in this species. NaArs is a commonly used elicitor of oxidative stress^20–22^. Arsenite, a common form of arsenic, impacts protein folding directly by reaction with thiols, but is also thought to stimulate ROS production leading to oxidative stress. Paraquat is an herbicide that causes tissue damage to a wide variety of organisms through its generation of the reactive oxygen (ROS) species superoxide after interaction with the mitochondrial electron transport chain^23^. Paraquat has been used to model the sequelae of oxidative stress in multiple species, including *C. elegans*^24^ and *D. melanogaster*^25^. To determine appropriate oxidative stress conditions, we performed dose response curves to determine appropriate concentrations of NaArs or paraquat for inducing acute oxidative stress. Based on our results, we chose to use 0.001% NaArs (Fig. 5A) and 1 mM paraquat (Fig. 5B) as the appropriate dosages to induce acute oxidative stress.

**Figure 6:**
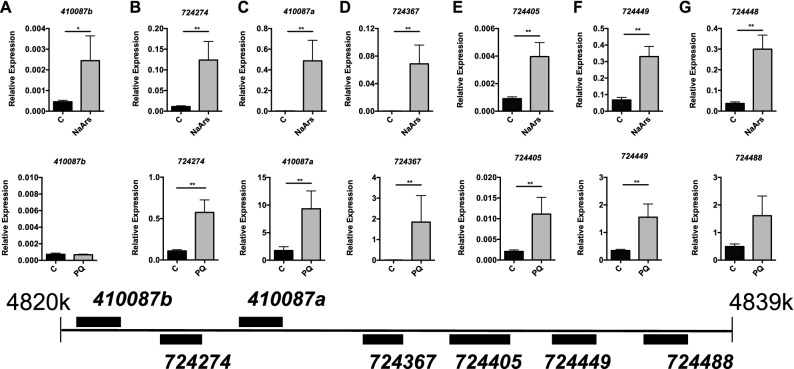
Select sHSP genes are induced during oxidative stress. Transcript levels of the *l(2)efl* genes *410087b* (**A**), *724274* (**B**), *410087a* (**C**), *724367* (**D**)*, 724405* (**E**), *724449* (**F**), and *724488* (**G**) relative to *β-actin* in midgut tissue from adult bees captured at the landing board and fed sucrose solution alone (C, n = 8) or with 0.001% NaArs (n = 8) (top row) or fed sucrose solution alone (C, n = 8) or 1 mM paraquat (PQ, n = 8) (bottom row) for 24 h. A schematic diagram of the colocalized *l(2)efl* genes in the honey bee genome is shown below for reference. Mean ± SEM is shown and represents expression values of the genes of interest calculated using the 2^(−ΔCT)^ method for individual bees. Statistical significance is noted as *p < 0.05, and **p < 0.01.

Using qPCR, we examined oxidative stress dependent induction of the seven sHSP genes in midgut tissue after 24 h. For NaArs we observed increased levels of transcripts of all seven after NaArs feeding (Fig. 6A–G, top). Statistics details can be found in Supplementary Table 6. Relative to *β-actin*, we observed increased levels of transcripts for *724274*, *724367*, *724405*, *724449*, and *410087a* (but not *410087b or 724488*) after paraquat treatment (Fig. 6, bottom). *β-actin* levels were similar iregardless of NaArs or paraquat or treatment as assessed by Ct values (Supplementary Fig. 5). Statistics details can be found in Supplementary Table 2.

### Predicted transcriptional regulation of *l(2)efl* sHSP genes in honey bees

Based on the diversity of stresses able to promote gene induction, we were interested in exploring the potential *trans* factors and *cis* elements responsible for regulating increased gene expression. Focusing on the four *l(2)efl* genes we found to be upregulated by all stresses, we sought evidence of conserved transcription factor binding sites (TFBS). In other systems, *sHSP* genes are regulated by a number of transcription factors, including HSF in both worms^26–28^ and flies^29–31^. Evidence of HSF-dependent induction of select *shsp* genes has also been shown in yeast^32^ and mammals^33^. We find evidence of canonical Heat Shock Elements (HSE, consensus sequence = GAANNTTCNNGAA^34,35^) at multiple points in these seven genes (Supplementary Fig. 6). We then examined these genes for evidence of direct regulation by UPR-activated transcription factors. Three *cis*-acting response elements have been characterized in the promoter regions of UPR-induced genes in mammals. These are the ERSE (ER Stress Response Element, CCAATN9CCACG), the ERSE-II (ER Stress Response Element II, ATTGGNCCACG), and the UPRE (Unfolded Protein Response Element, TGACGTGR) (Reviewed in Ref.^36^). While these sites were not evident, there were a number of instances of another ATF4-binding motif, TTKCATCAK that has recently been characterized in mammals^37^ and shown to regulate gene expression in insects^38^ (Supplementary Fig. 6). The transcriptional regulator controlling the oxidative stress response, NRF2 (and its homologs), are responsible for promoting resistance to oxidative stress in *C. elegans*^39^,* D.* melanogaster^21^, and mammals (reviewed in Ref.^40^). The Nrf2 signaling system is functionally and structurally conserved in *D. melanogaster*^41^, which possess both a Nrf2 homolog, the Cap'n'collar splice form C (CncC) protein, and the E3 ubiquitin ligase that regulates it, Kelch-like ECH-associated protein 1 (Keap1). Honey bee possess genes encoding CncC (LOC725081) and Keap1 (LOC411679). Nrf2 responsive genes possess the consensus binding site (TGAYNNNGC) which makes up the core of the antioxidant responsive element (ARE)^21^. We found a number of these TGAYNNNGC sites near these four genes (Supplementary Fig. 6). In addition to NRF2, the transcription factor FOXO has been shown to be an important regulator of oxidative stress responses and can upregulate transcription of sHSP genes in both worms^26–28^ and flies^29–31^. We observed a number of consensus sites for FOXO binding (TKTTYACY^42^) at multiple points in these four genes (Supplementary Fig. 6).

### LAMP assay detection of *l(2)efl* sHSP genes

**Figure 7:**
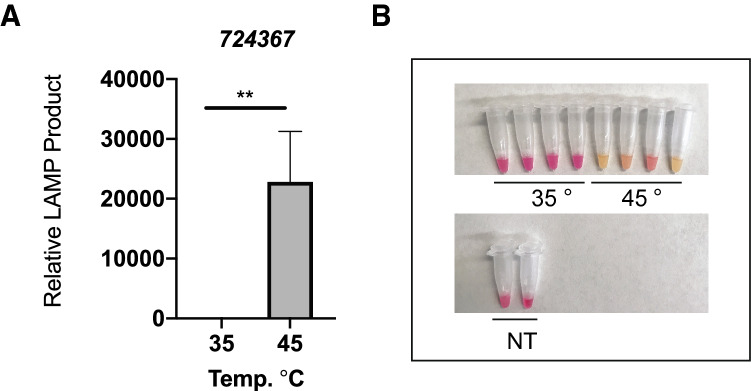
RT-LAMP assay detects heat-induced induction of the *724367 l(2)efl* gene. Transcript levels of the *l(2)efl 724367* gene in midgut tissue from adult bees captured at the landing board and maintained for 4 h in cages at either 35 °C or 45 °C as assessed by RT-LAMP using quantitative real-time fluorescence (**A**) or end-point colorimetric (**B**) read-outs. For real-time fluorescence, mean ± SEM is shown and represents relative expression values of *l(2)efl 724367* (see “Methods” for details). Statistical significance is noted as *p < 0.05, and **p < 0.01.

We developed an RT-LAMP assay to measure the relative amount of transcript from the *724367 l(2)efl* gene. We chose this gene as it is induced by the most stressors and demonstrates the highest level of induction. We observed that our RT-LAMP assay can detect increased *724367* expression in the midguts of bees heat shocked at 45 °C for 4 h when compared to bees maintained at 35 °C for that period using real-time fluorescence relative quantification (Fig. 7A, p = 0.0002, t = 8.742, df = 5.456). We also found that we could easily differentiate samples from control or heat-shocked bees using visual assessment of an end-point colorimetric read out (Fig. 7B).

## Discussion

As no single cause for the recent increase in honey bee disease has been found, there is intensified focus on the synergistic impact of disparate stressors working in concert. A critical first step in understanding such combinatorial impacts involves defining specific common cellular processes that are impacted by multiple stressors and could consequently serve as links to cellular dysfunction, tissue pathology, disease, and mortality in honey bees^43^. The various pathways that make up cellular responses to disruptions in proteostasis represent one set of processes to examine for such interactions. We used an unbiased approach to identify novel genes altered in expression by induction of three proteostatic network responses in the honey bee using transcriptome profiling (RNASeq)^6–8^. We hypothesized that we could then utilize this information to better understand how these stresses interact to impact honey bee health and to identify candidate biomarkers of stress in honey bees. Here we report that genes encoding sHSP, sometimes known as the ‘first line of defense’ against protein folding stress, (reviewed in Refs.^14–17^), are part of a common cellular response to a broad array of stressors. In other invertebrates, such as *D. melanogaster* and *C. elegans,* sHSP are involved in maintaining proteostasis in the face of diverse environmental insults. *D. melanogaster* possess 12 sHSP-coding genes^44^. Ten of these have been shown to be upregulated by heat stress, while seven are affected by oxidative stress. Notably, the single *D. melanogaster* (*l(2)efl)* has been shown to be part of a core stress response to diverse stresses along with HSP70 and HSP83^45,46^. *C. elegans*, contains 16 genes encoding sHSP. As with those in *D. melanogaster*, some genes are inducible by stress, while others are not (reviewed in Ref.^47^). Outside of the highly conserved α-crystallin domain, these proteins display significant areas of low homology in the N-terminal and C-terminal regions and range in size from 12 to 42 kD.

With some exceptions, our phylogenetic analysis of the sHSP proteins in other bee species shows a high degree of conservation of this family among bees from different life histories. Examination of the underlying gene structure also shows striking conservation of the *shsp* gene cluster. However, *shsp* number and gene structure do not obviously correspond to the known phylogenetic relationships among bees^19^. For example, *M. genalis* and C*. calcarata*, which are both found along with *A. mellifera* in the Apidae family, have reduced numbers of shsp genes and a remarkably divergent gene structure while *M. rotundata* and *O. lignaria*, which are both in the Megachilidae family, share *shsp* gene number and structure with *A. mellifera*. Due to their critical role in pollination in natural ecosystems, there is great interest in understanding the factors influencing the health of non-Apis bee species^48^. Thus, future work to better understand the evolutionary context for these differences and to define whether various bee species have different stress responses and tolerances will be important. In addition, while not currently, validated, our results suggest that *shsp* may provide potential biomarkers of stress in some non-Apis bee species.

In other model systems, studies of diverse stressors and their cognate response pathways has uncovered both unique and common gene targets. In *D. melanogaster* for example, heat shock, oxidative stress, and ionizing radiation all upregulated a core set of shared genes, although unique gene targets were associated with each stress trigger^45^. Multiple studies have also shown that interactions between stress pathways are often complex. For example, the OSR is inhibited by heat stress in *C. elegans*^49^ while it synergizes with the UPR^50^. In *D. melanogaster*, *l(2)efl* has been shown to be part of a core stress response to diverse stresses along with HSP70 and HSP83^45,46^. In other organisms, *sHSP* genes have been shown to be regulated by a number of transcription factors, including HSF (in both worms^26–28^ and flies^29–31^) and FOXO (in both worms^26–28^ and flies^29–31^). Our TFBS analysis suggests that the honey bee *sHSP* genes located in the cluster are potentially regulated by HSF, ATF4, NRF2, and FOXO, likely acting in concert. However, future studies will be required to determine the functional importance of these TFBS as the sequence motifs responsible for the binding of most transcription factors have not been empirically tested in the honey bee.

Our work is the first report of sHSP genes being transcriptionally upregulated by the UPR. The cellular benefit of increasing cytoplasmic sHSP protein production in the face of UPR (and ISR) is not clear. For the UPR, triggered by unfolded protein build-up in the ER, it is possible that increasing sHSP expression in the cytoplasm is important to help respond to the increased transport of these unfolded proteins to the cytoplasm for degradation by the ERAD pathway. For the ISR, activated by halofuginone or cyclohexamide, translational pausing might be expected to reduce the typical burden of unfolded proteins in the cytoplasm. However, the ISR and UPR all result in reduced protein synthesis. This impact of inhibition on different proteins is uneven and may contribute to stoichiometric differences that disrupt the assembly of protein complexes resulting in the need to protect against aggregate formation. Such issues have been observed during mito-nuclear protein imbalances caused by disruption of mitochondrial protein synthesis^51^.

As declining health in a honey bee colony is often caused by interacting stresses, being able to quantify individual stressors such as infectious agents or pesticides in and around colonies is critical for monitoring possible exposures to disease-causing agents. However, for identifying resultant unhealthy colonies, the ability to measure the relative stress of individual bees or the colony as a whole through the use of biomarkers represents a key diagnostic tool^48^. Quantification of cellular stress responses, often through measuring of HSP, has been used as a surrogate to identify general organismal stress in a variety of settings, including the honey bee (reviewed in Ref.^3^). Based on the robust induction of some of the *l(2)efl* genes in response to a broad array of stressors, quantification of these sHSP may provide an optimal biomarker for honey bee stress. Recently, another group proposed sHSP of the *l(2)efl* family as potential biomarkers for honey bee queen health after they were identified using a proteomics discovery strategy^52,53^. In their study, these authors found that the proteins encoded by the *l(2)efl* genes *724367* and *412197* had some limitations for predicting stressed bees. This group also discussed a number of key aspects of diagnostic testing that will be critical to explore in future work, including the dose-responsiveness of the potential biomarker and the time-course of induction after exposure. Other aspects to consider include factors that may impact stress responses such as age or caste. Some of the sHSP genes discussed here have been shown to be part of the antiviral response in honey bees and bumble bees^54,55^. Finally, as a new biomarker used to measure individual bee stress, the levels of *l(2)efl* gene expression have not yet been linked to colony-level outcomes. As an example, the expression of yolk protein Vitellogenin and select immune genes (as well as abdominal lipid levels) were used to link individual bee health to colony health and survival in a seminal biomarker study^56^.

For biomarker detection, a point-of-care testing (POCT) assay would be the most useful. Future progress in a number of key areas would be necessary for making any new POCT strategies both desirable and feasible for use by beekeepers in the field. First, sample preparation methods that are simple, inexpensive, and provide an adequate sample size need to be established. Second, a rugged assay that consistently provides reproducible and accurate results is critical. Third, robust and easy to use hardware and software that maximize user benefits and promote adoption must be developed. Finally, any new assays need to be compared to the current gold-standard diagnostic tools and coupled with empirically derived management recommendations. LAMP assays have many features that make it ideal for POCT analysis, including simplified sample preparation potential and fast reaction times using inexpensive equipment (reviewed in Ref.^57^). However, the hardware and software used to run and analyze assays still represent limiting factors. In recent years there has been burgeoning interest in coupling diverse POCT strategies with the smartphone as a measurement and analysis platform^58^ due to its ubiquity and versatility^59^. Attempts to couple LAMP with detection using smartphone optics have been quite successful in other settings^60–62^, allowing for inexpensive quantification and analysis of results. Since the advent of COVID-19, there has been explosive progress in both the assays^63^ and the specialized accessories^64^ required for such assays, making this the ideal time to pursue LAMP-based strategies for diagnosing honey bee disease more broadly. The ability of recent developments in 3D printing technology to facilitate advances in POCT are particularly exciting^65^. For example, Nguyen et al. designed and fabricated a single accessory that can perform the heating required for the LAMP reaction and assist in the imaging using a smartphone^66^.

Transcriptome profiling identified a subset of sHSP of the *lethal (2) essential for life* (*l(*2*)efl*) gene family as common transcriptional targets activated by diverse cellular stressors. Interestingly, this work is the first report of sHSP genes being transcriptionally upregulated by the UPR and ISR. Our data suggests that *l(*2*)efl* genes are part of a core stress response that might provide an effective biomarker for cellular stress in honey bees. We designed and tested an RT-LAMP assay to detect increased *l(*2*)efl* gene expression in response to heat-stress, thus providing a powerful proof of principle for using *l(*2*)efl* gene expression as a biomarker of stress. Future work will be necessary to link changes in *sHSP* gene expression to colony-level outcomes, to adapt our preliminary assay into Point of Care Testing (POCT) assay appropriate for use as a diagnostic tool for use in the field, and to couple assay results to management recommendations.

## Supplementary Information

Supplementary Tables.

Supplementary Figures.

## Abbreviations

HSR: Heat shock response
ISR: Integrated stress response
OSR: Oxidative stress response
UPR: Unfolded protein response
sHSP: Small heat shock protein
