# Supplementary material for: Honey bee sHSP are responsive to diverse proteostatic stresses and potentially promising biomarkers of honey bee stress
Source: Sci Rep. 2021 Nov 11;11:22087. doi: 10.1038/s41598-021-01547-1 (PMC8586346; doi:10.1038/s41598-021-01547-1)

Supplemental Figures

Supplemental Figure 1. New mRNA and protein models for *Apis mellifera LOC410087* gene

mRNA Sequences:

>410087a PREDICTED: protein lethal(2)essential for life-like [Apis mellifera]
AATTTATTGCAATATGACTTCGTACATTTCTTCTATTCTAATAATGTATTCTAAATAATTTTAAATCGTATAATAACATTCT
AAAGTTTTTTTTTTTTTAAATATATTTATTTTTATCTTTCTTTTAAATTTTTTCAAGGAAATATTAACGATAAAGAAATAAC
AAATAGAAAAAATTTAATTCTCGCTCGAAAATATTTTGCTTTTTTTTTATTAATTTGTAACTATAAATAATCATGACATAT
AGATATGACGTTACGTCATTGATCGATTTAGAAATTTCTAGAAAGATCCAAAAAATATTCTAGAATTTTCACGAAAATTT
CAGTCGTCATTTCACGCGCGTCTAAAGACCCCCTCATTGAGTATATATAAAGTTGCGCTGTCGGCATACGCTGTCAGTA
TACAGAGATCGAGTGACGAGTGCGCACGTATTTCCGCATTGATAACAAAGTTAATTCTTTCAAGAAAGTGTGAAGAAA
AGTAGAAATTCAAAatgaggagaggaatgacattaattccaagattgttttcccattggtgggaagcattggaacaaccacatcgattattggatcaa
cattttggaagaggattgcgagcagatcaactttttccttcgataccattcagatcatttccttataatttttctcgaccatggatagattgggaacgagagga
agattgtggttggtccataatgagaaatgacaaagacaaatttcgagtgattcttgacgtgcagcaattcaaaccagaagaaataaatgtcaaagttattg
acaatttcattgttgttgaaggaaaacatgaagacaaggcagatgatcacggtttaatctccagacacttcgttagaaaatatttagtaccagatcaatgtg
atcctgagaaagctgcaagtagtttgtctacagatggtattttaacgataacagcaccattaagacctgaagctgctgaaagtaaacgggaaaaaactata
aaaatcgaacaaacggggaaacctatggtagaagatgaacctgaaaaaataaaacaaacacaatagGAAATGAATTAATTTTGTACATTTA
GTATTTTTGTGTGTTTTTATTTTTTACTGTATTCATTAGTGTACAGATAATTATAATTATTTTTATATATGTATATATATAT
ATATTCGAAAGATGTTTGTTTGTGATGAAGAATAAATGATATGTAAATATATTATAAAAAATAGTGTAATATTTCAGTA
TTACTTATACAACCTATATTCCTTGTACTAAATTTTAATTAATTACAAAGAATTCTTTATTAATACGTATACGTTTTGCATA
TATACATATACATTACATATACATTTTATTTGTTTAACAGAGATTATTTTAATATGTATTTCTTTTTTAAGTGCAAATATTG
TAATAAGAATTTAATACCATTTAAATTTTATTATTTAAATTTAAAATTTTGATATCATTGATTAATTATCTATTTTATAAAT
TCTTATGAATTTTTTAATAAATGTGCTAAAACTTTTAAGAATGGAAGGAAGAAAAATATATTAGAAAATATAATATTAA
AAATTATAATATTA

>410087b PREDICTED: protein lethal(2)essential for life-like [Apis mellifera]
AAATCTTATATATAATATTTTATAAGATTTTTTTAATGTAAAATGAATTATTATGTAAAATTAATATATGTATATATATTA
TACAATATATTTTATAGAATAGAAGATTCATGAAGATTCAAAATTATACAATTTTATTTACATGAGCTAGTTGCTAATTG
AATGTTCGCGCTTCTTTAAGCAGATAGAAACAATATTTTAAAATAGTTCTTATTTTATTTAAAATAATTAATTAAAAGTTA
AAAATTAATATTTTGGCAATTAAAATCTTTCTATTTTCTATTTAGAAAATTCGATTTCCAGAAAACTCTATATGTTTCTTG
ATTATCGTTACCCCCACTTTTCATGGCGTATGAAATATATATTCGCGCGTAGGAATCATTATAATAGAATTCGAACGAA
ACTATTGCATAAACATTTAGCCAAACATCTTATTGTAGTCAAGCAGTACGTGAGTGAATAAAAAAGCTTTATAAAAGAA
AAGAAAATTTTAAGTGAATTATTTTATATATATATTTTTATTATATATATAGATTAAATTTATTTTGAAATTAAAAGAAAA
ACAatgtctttttttgccggtacttcttaactggggggaggatttgaagacaccttattatccctttgaccagcttcttggacttccattgagttctgaagatttat
caacatctttctttcctgacgatactgatattttaatgctaagacctcgtcgctgttttcgcagatatcagccttataaaagaattatagatcgtacatccagtg
gaacatctacaatacaagctgataaaaataaatttcaagtatcattagatgttcaacaatttgctccagaagaaatgactgttaaagttgtaggaaagaatg
tagttgtcgaaggcaaacatgaagaaaaacaggatgaacatggatggatatccaggcattttgtacgaaagtatattgtaccagaacaatgtgatattgat
caattaaaatcaaatttatcttctgatggaatattgatgatcactgcaccaagaaaagagattgatccaacatcaaagaatgaaagaattattaaaattcaa
attacaggaaaacctgctttaagagatgatacaaaaccaattgaaaaacaaaaggaaaatcaagttcagaagaatcccatatcacaaagaggacaaga
acatatagtaaaagctgcttagGAAAATAATTTATTGTAAAATTGTTATAACAACAATTTTCTTAGTATTTTATGTTGCATATAT
AATTAACTTAAAGCTCAACTAAATTCTTTATCTAAATCTATCTTATATTTTCTAATAAAATAAGCAATTTAATTTTAATTTT
TCATATTTTAAATTTAAGTGTGATCTAGTTGAATAACAAACTAAATTTGTAAAATAAACATTTCTCATTTTTATAAGTTAT
TCATATATTATTTAAGATTCATTTGTCATAAACATGTTTTTTAATAAAACTGTTACTGTTTTTGTATAATTTTAAATTAAAT

ATATATATCCCATAATTTTATAATTTCATGTATCATTTTCAAATATAAATAAATATAACAAATTTTAAGATAATATTTTTAT
TGGATTATACATATAAAAATTAACATTTAATATTTAATAAATTTAAAAAATTATTTTTTTATTTAACAATGGACGGAATTT
TAATATATATCTATTAAAGAAATTTAAAAATATTTATATTATTGGCTGATGAAAAAATAGTAAATTTAGTAATATATATT
ATATATAAATTAAGCAATTTAGTTTTAATTTTTCAATACAA

Protein Sequences:

>410087a PREDICTED: protein lethal(2)essential for life-like [Apis mellifera]
MRRGMTLIPRLFSHWWEALEQPHRLLDQHFGRGLRADQLFPSIPFRSFPYNFSRPWIDWEREEDCGWSIMRNDKDKFRVI
LDVQQFKPEEINVKVIDNFIVVEGKHEDKADDHGLISRHFVRKYLVPDQCDPEKAASSLSTDGILTITAPLRPEAAESKREKTIKI
EQTGKPMVEDEPEKIKQTQ

>410087b PREDICTED: protein lethal(2)essential for life-like [Apis mellifera]
MSFLPVLLNWGEDLKTPYYPFDQLLGLPLSSEDLSTSFFPDDTDILMLRPRRCFRRYQPYKRIIDRTSSGTSTIQADKNKFQVSL
DVQQFAPEEMTVKVVGKNVVVEGKHEEKQDEHGWISRHFVRKYIVPEQCDIDQLKSNLSSDGILMITAPRKEIDPTSKNERI
IKIQITGKPALRDDTKPIEKQKENQVQKNPISQRGQEHIVKAA

Supplemental Figure 2. Schematic of the gene organization of *l(2)efl* genes in select bee species (both genes found in the cluster as well as genes found outside).

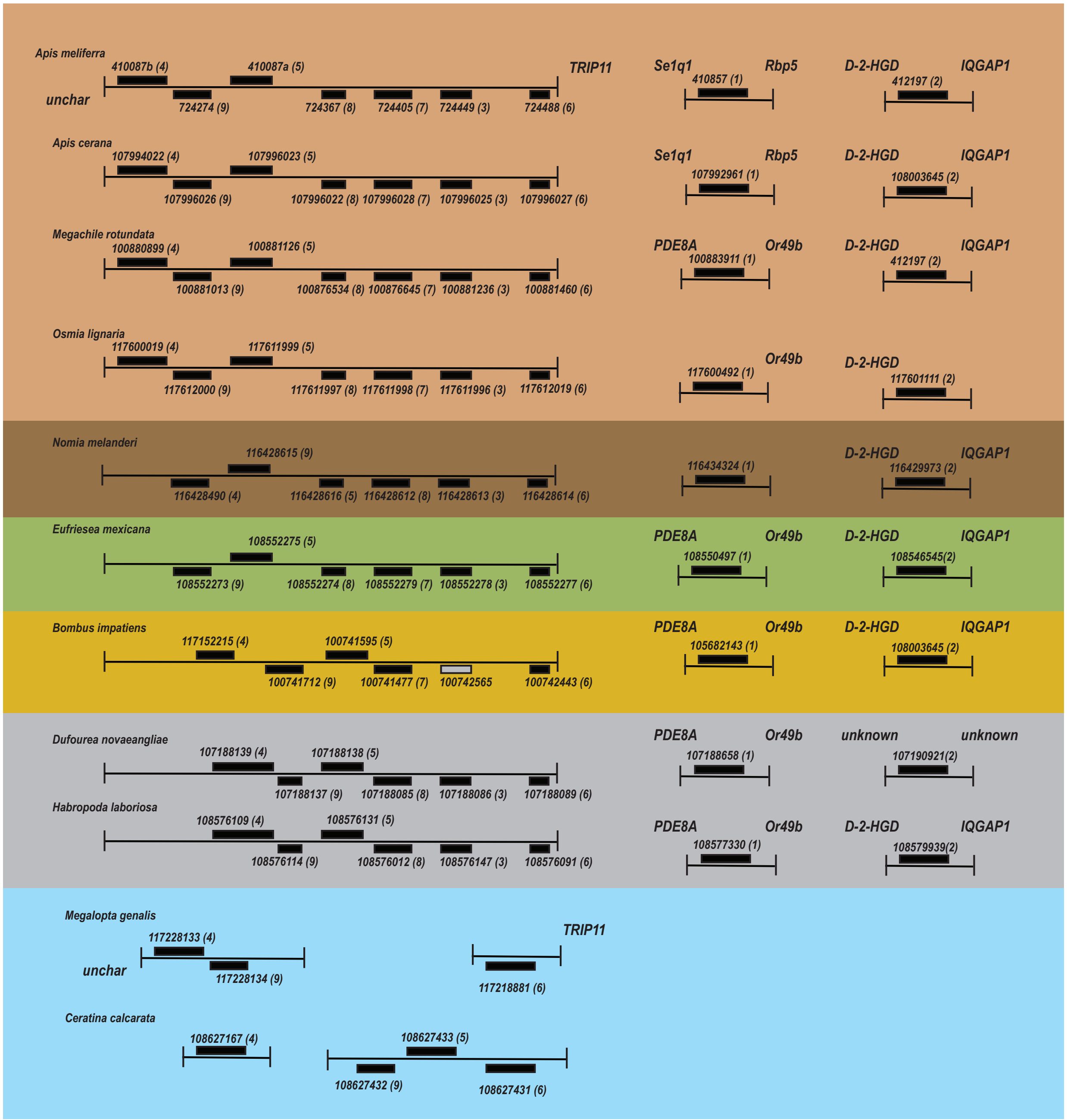

Supplemental Figure 3. Select sHSP genes are induced during Heat-Shock. Transcript levels of the cluster *l(2)efl* genes *410087b*, *724274*, *410087a*, *724367, 724405*, *724449*, *724488* and the non-cluster *l(2)efl* gene *412197* after heat shock (C) relative to *β-actin* in head tissue (predominantly brain and sensory organ tissue), midgut, thorax tissue (predominantly flight muscle), and abdominal wall (predominantly fat body) from adult bees captured at the landing board and maintained for four hours in cages at either 35 ° (n=6) or 45 °C (n=6). Symbols represent expression values of the genes of interest calculated using the $2^{(-\Delta CT)}$ method for individual bees. Mean ± SEM is also shown. Statistical significance is noted as $*p < 0.05$, and $**p < 0.01$.

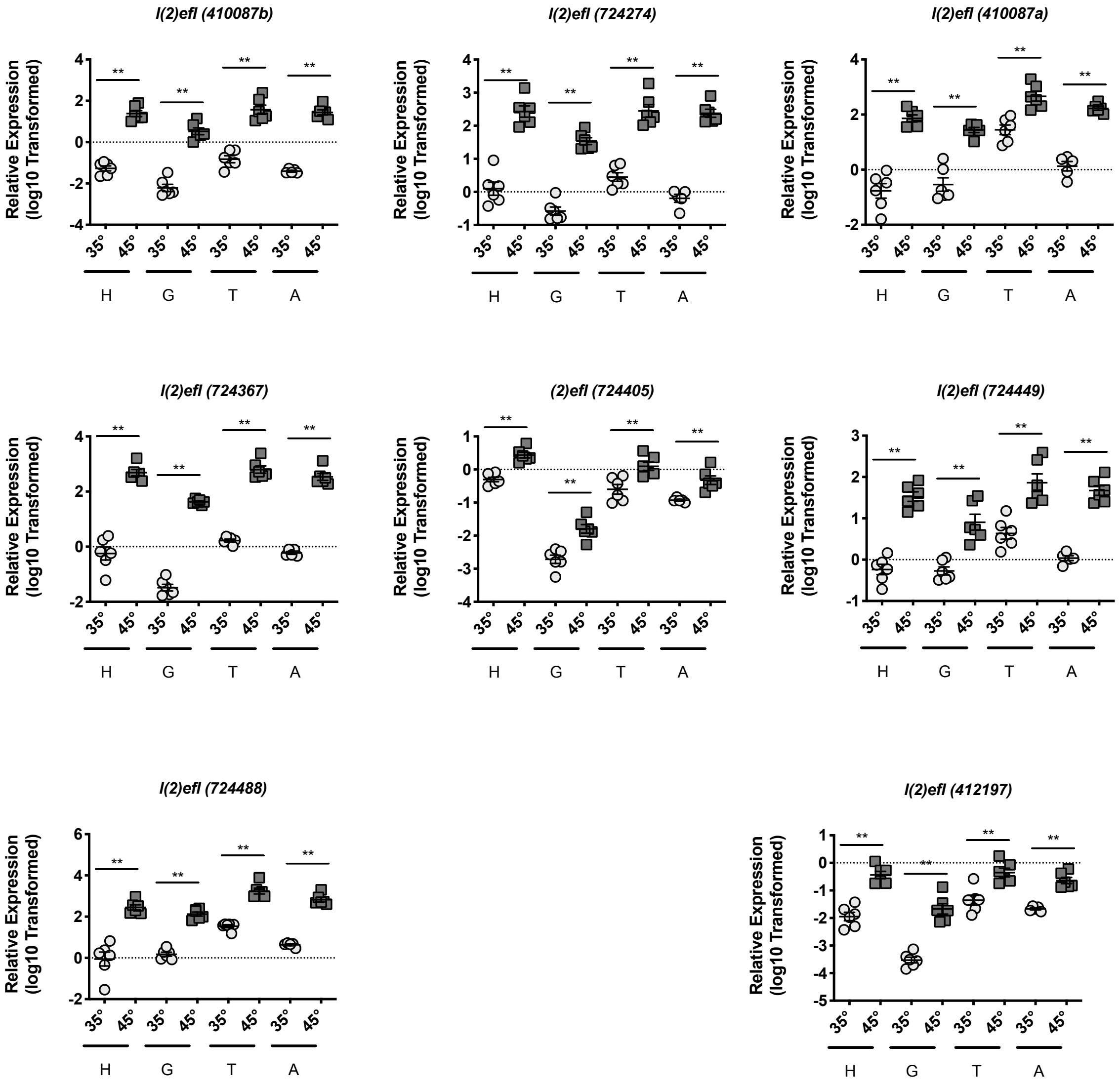
l(2)efl (410087b)
l(2)efl (724274)
l(2)efl (410087a)
l(2)efl (724367)
(2)efl (724405)
l(2)efl (724449)
l(2)efl (724488)
l(2)efl (412197)
Relative Expression
(log10 Transformed)
35°
45°
H
G
T
A
**

Supplemental Figure 4. Transcript levels of putative *Hsp68* (*Hsc70-4*) (A), *l(2)efl (410857)* (B), and *Heat shock protein beta-1 (Hspβ1)*(C) relative to *β-actin* in head tissue (predominantly brain and sensory organ tissue), midgut, thorax tissue (predominantly flight muscle), and abdominal wall (predominantly fat body) from adult bees captured at the landing board and maintained for four hours in cages at either 35 °C or 45 °C. Mean threshold cycle (Ct) for *β-actin* (D) in these tissues. Symbols represent expression values of the genes of interest calculated using the $2^{(-\Delta CT)}$ method for individual bees. Mean ± SEM is also shown. Statistical significance is noted as *p < 0.05, and **p < 0.01.

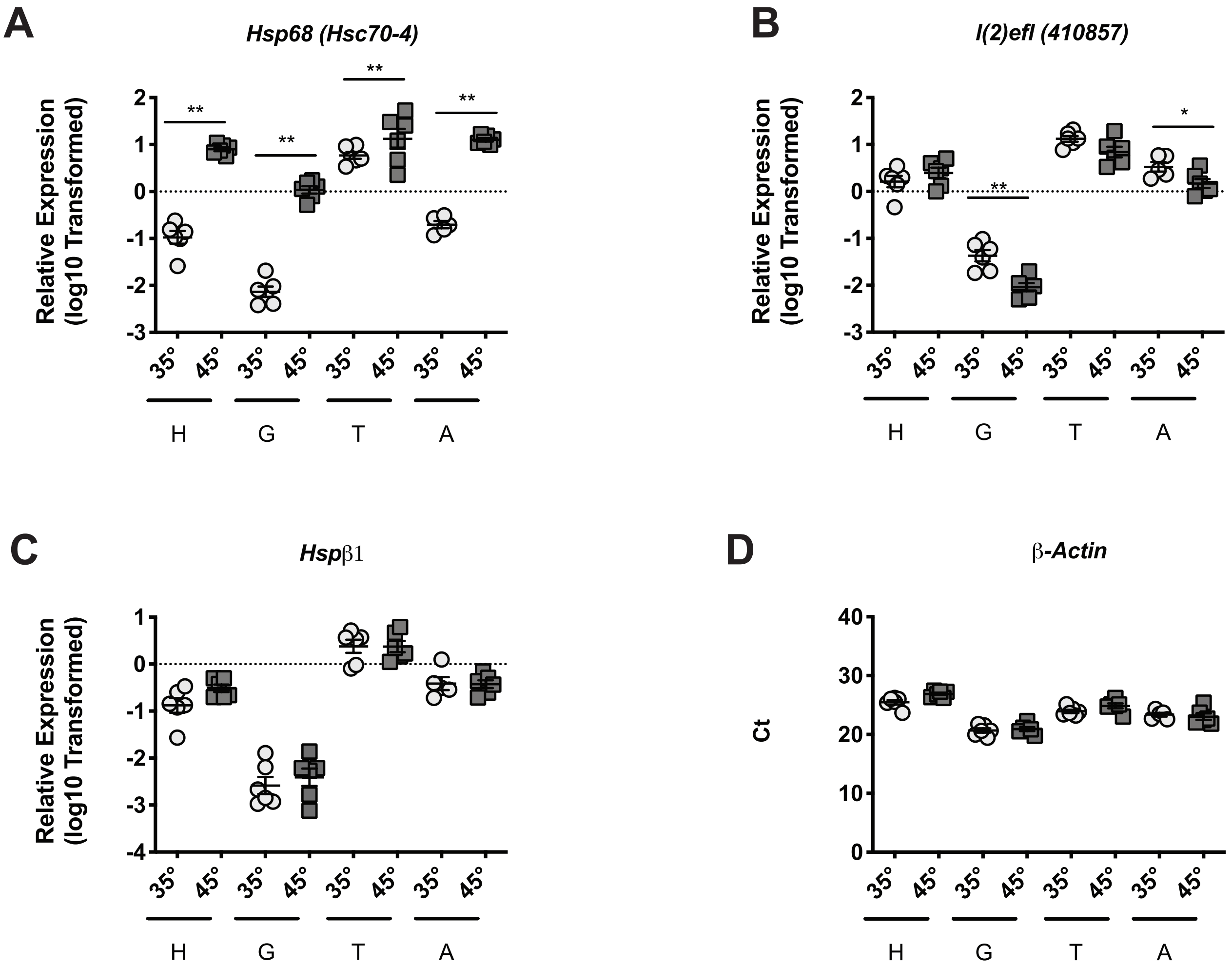

Supplemental Figure 5. Threshold cycle number for *β-actin* from the bees from experiments with tunicamycin (A), halofuginine (B), paraquat (C), NaArs (D), and cycloheximide (E).

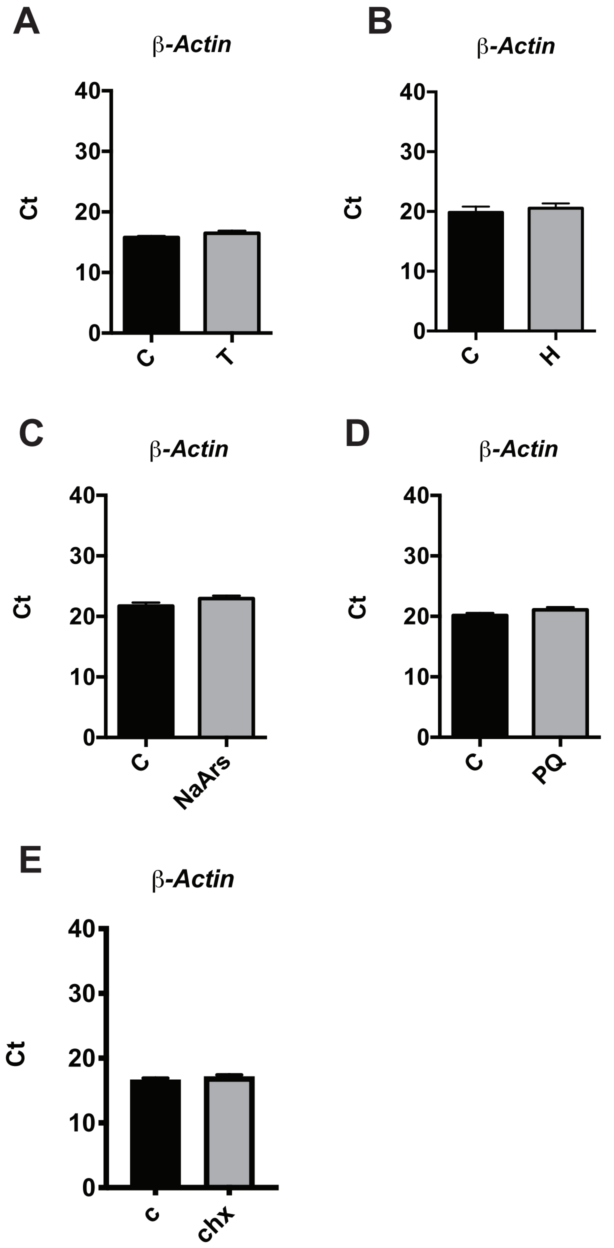

Supplemental Figure 6. Sequence and annotation of *l(2)efl* genes located in the cluster with key predicted transcriptional factor binding sites. Underlined regions represent transcribed regions. Lower case letters denote open reading frames. Bolded genes are in the sense direction while non-bolded genes are in the anti-sense direction.

HSE (Heat Shock Element) consensus sequence = GAANNTTCNNGAA
FOXO binding = TKTTYACY
UPRE (Unfolded Protein Response Element = TGACGTGR
ATF4-binding motif = TTKCATCAK
AREs (for antioxidant response element) = TGAYNNNGC

TTGTATTGAAAAATTAAAACTAAATTGCTTAATTTATATATAATATATATTACTAAATTTACTATTTTTTCAT
CAGCCAATAATATAAATATTTTTAAATTTCTTTAATAGATATATATTAAAATTCCGTCCATTGTTAAATAAAA
AAATAATTTTTTAAATTTATTAAATATTAAATGTTAATTTTTATATGTATAATCCAATAAAAATATTATCTTA
AAATTTGTTATATTTATTTATATTTGAAAATGATACATGAAATTATAAAATTATGGGATATATATATTTAATT
TAAAATTATACAAAAACAGTAACAGTTTTATTAAAAAACATGTTTATGACAAATGAATCTTAAATAATATATG
AATAACTTATAAAAATGAGAAATGTTTATTTTACAAATTTAGTTTGTTATTCAACTAGATCACACTTAAATTT
AAAATATGAAAAATTAAAATTAAATTGCTTATTTTATTAGAAAATATAAGATAGATTTAGATAAAGAATTTAG
TTGAGCTTTAAGTTAATTATATATGCAACATAAAATACTAAGAAAATTGTTGTTATAACAATTTTACAATAAA
TTATTTTCctaagcagcttttactatatgttcttgtcctctttgtgatatgggattcttctgaacttgatttt
cctttgtttttcaattggttttgtatcatctcttaaagcaggttttcctgtaatttgaattttaataattct 410087b
ttcattctttgatgttggatcaatctcttttcttggtgcagtgatcatcaatattccatcagaagataaattt
gattttaattgatcaatatcacattgttctggtacaatatactttcgtacaaaatgcctggatatccatccat
gttcatcctgtttttcttcatgtttgccttcgacaactacattctttcctacaactttaacagtcatttcttc
tggagcaaattgttgaacatctaatgatacttgaaatttatttttatcagcttgtattgtagatgttccactg
gatgtacgatctataattcttttataaggctgatatctgcgaaaacagcgacgaggtcttagcattaaaatat
cagtatcgtcaggaaagaaagatgttgataaatcttcagaactcaatggaagtccaagaagctggtcaaaggg
ataataaggtgtcttcaaatcctccccccagttaagaagtaccggcaaaaaagacatTGTTTTTCTTTTAATT
TCAAAATAAATTTAATCTATATATATAATAAAAATATATATATAAAATAATTCACTTAAAATTTTCTTTTCTT
TTATAAAGCTTTTTTATTCACTCACGTACTGCTTGACTACAATAAGATGTTTGGCTAAATGTTTATGCAATAG
TTTCGTTCGAATTCTATTATAATGATTCCTACGCGCGAATATATATTTCATACGCCATGAAAAGTGGGGGTAA
CGATAATCAAGAAACATATAGAGTTTTCTGGAAATCGAATTTTCTAAATAGAAAATAGAAAGATTTTAATTGC
CAAAATATTAATTTTTAACTTTTAATTAATTATTTTAAATAAAATAAGAACTATTTTAAAATATTGTTTCTAT
CTGCTTAAAGAAGCGCGAACATTCAATTAGCAACTAGCTCATGTAAATAAAATTGTATAATTTTGAATCTTCA
TGAATCTTCTATTCTATAAAATATATTGTATAATATATATACATATATTAATTTTACATAATAATTCATTTTA
CATTAAAAAAATCTTATAAAATATTATATATAAGATTTTTATTTTGTTATATTATATTATTTTTATACTTTGA
CAATATATTGTAAAAAATGTATAAAAATTGTGTTTAAATGACTTAAAAAAATACTTAATGTTTTATATAATTT
TAAACAAAATTTCAATATTATTTTAATTAAAATTTGACTATTTTCATCATTGACAATTTTGTTAAAATCAAAT
TTCAATAGTTATAAACATATTTATTTAAAGAAATATTTTATTTCCAATATTTCTTGACTTTATGATAATGCTA
TTACATCAAACAAGAATAAATTATTGAAATTAAAATCAAAATTAAATTAATCAAATAACAATTATTAATAGTT
AATAATTTTAAATTTAGAAACTATATTTTTTTAATAAAAAAATATTAAATAATTGATAAATGAATTAATTTTC
TTGAAATTTATAAGAATTTATTTGAAATTTGTGATATATTCAATAACTAATTAATATTCATTTTTATATATCT
TTTGATTTACAATTTTAAATGTTTTATGATAGTTTATTTTTTTAATCTCTTTTATTTTTCTAATCTGATCTAA
CTGAATATATTTCCAGTTAAAAAATTTTCTCTACTGAATTTTCTAGAAATAATACATATGTATTAAATAAATC
CATTATATGTAATCATCATATATACATAAAATTTACATATTTAATAAACAATTTTGAAGAAATTACTATCATT
ATACTATCATTACTATCATTATTATAAAAATCTTAAATTTAAGTAAGATGAAAAATGTTTATATATTTTTATA
GAAACTTTTTTTTTTTTTTCAAATAAATTATATGTAATGTAAATATTATAGTACTAAATTTATTAATAAATTT
TAAATAATATTATTGTACAGTTTATTGTAAACTATATTTAACTTAAATACTTAAGTATATTATATTTAAATGA

AAATGCAAGTTATTTATTTAATTCTTATCATAGATATTTGAATTATATACGTACATATTTTATAATATGAAAA
ATTTTAATTTCACATATAATAAACTCTCTCTGACTGTTAACCTAGATATACATATGTATGTATTATACAGTGT
CCACTCAATAGTTGCTCCGTTCTCGAATTCTCTTGAAGTTTCGCGACTGCGCAAATTCATGTATAAATACGGC
CG**ATAAGAACCGCTATCGTCAGTATTTTGTTAGCAATCAAACAGTGAAGATCGCAAGTTTGAGTGAATTTCAC**
TCAATTTCACTAGATAAAAGCATTAGACACTATTAATACATTTGTTAAATTAAATTGTCGCAAAatgtcattg 724274
gtacctttgttattctctgattggtgggagactctggatcgcccgcaccgcctcttggaccagaatttcggat
taggcctttatccggaacaattgttatcaccgagccgattggagttatgtttacaaccacgaagaggaaacgt
ttatatctccagaccgaattgggctgaactgtttcgtggtgacagaggcagttcaactgttcaggcagataaa
gataaattccaagttgtacttgacgttcaacagtttgaacctcatgaaattgatgttaaggtcgttgataaat
ttgtcgttgttacggcaaagcatgaggaaaaacgcgacgagcacggttggatttcacgtcagttcgtcagaaa
atatctgattccagagcaatgtgatcttgaacaagtctcatcgaagttatcatctgatggcgttctcactatc
actgctccaaggaaggatcaaggaaatgtagagaatgaacgagtaatcaaaattgagcaaaccggtaaaccag
caatccagacgaaaccacctaaacagcagcagaatcaaaatgaagaaaacgaagagaagaaataaAAGTTTCA
ATTAGATATTTCTGCCATTTATGAAATTTCCTAATACAATTATTATTGTTGAATTACAATTAAATTCCTATCG
TAACATTTAAAATTATTATAGTATTATTAAAAATGTGATGTAATATATATTGTGAATAGTTATTTTATTTTTT
TCATGTATATTATTTTCATAACTACGTAAATATATAAATATATATATATATATGTATATGTTACATAATTATT
TCTGCCAATATAGATTTATAAGATTTATGTAATTTTTTTTTATGTTTTAATAGATTTGTAATTAAATATTATA
TTTATTTTTTGTGAATTTTATCTTCTTATCATTCCTAGTTTTCAATTCTTTCTATTGAAAATCTGAACATAAC
TTTAATTCCTAAACTTTTGAAAATTATAACAAATATAATAACAAGTTATAATAAGATACATATCTTATTTGAA
AATCTTTTAATAAAAAATATCAAATTTTAGCATGTAATTAAAAATTCGTTATTGTATTATTTAAAAAATAAAC
ATTATCCAAAATACATATTATTTGTAATTGTTAATAAAAACCATTGCAAATATTATTCTTTAATTAAGATTAG
AATTCTTAGCACGCACAATGTTTGTGCATAATATTTTTTTTAAAGAATTTTTTCTTTAAAAAAAAAAAAAATC
TAAGAGTTAATTTCTTTTATGAATTTCAAAAATATAATATGTAATGTCAAATTTCATGATAATATATACACAA
GGAACGATATCACAATTTAGAAATATTATTTGATTTCAATAGATGTACAAATATAAATATAAATCATGTTTAT
TAATTATAATATTATAATTTTTAATATTATATTTTCTAATATATTTTTCTTCCTTCCATTCTTAAAAGTTTTA
GCACATTTATTAAAAAATTCATAAGAATTTATAAAATAGATAATTAATCAATGATATCAAAATTTTAAATTTA
AATAATAAAATTTAAATGGTATTAAATTCTTATTACAATATTTGCACTTAAAAAAGAAATACATATTAAAATA
ATCTCTGTTAAACAAATAAAATGTATATGTAATGTATATGTATATATGCAAAACGTATACGTATTAATAAAGA
ATTCTTTGTAATTAATTAAAATTTAGTACAAGGAATATAGGTTGTATAAGTAATACTGAAATATTACACTATT
TTTTATAATATATTTACATATCATTTATTCTTCATCACAAACAAACATCTTTCGAATATATATATATATACAT 410087a
ATATAAAAATAATTATAATTATCTGTACACTAATGAATACAGTAAAAAATAAAAACACACAAAAATACTAAAT
GTACAAAATTAATTCATTTCctattgtgtttgttttatttttttcaggttcatcttctaccataggtttccccg
tttgttcgattttatagtttttcccgtttactttcagcagcttcaggtcttaatggtgctgttatcgttaa
aataccatctgtagacaaactacttgcagctttctcaggatcacattgatctggtactaaatattttctaacg
aagtgtctggagattaaaccgtgatcatctgccttgtcttcatgttttccttcaacaacaatgaaattgtcaa
taactttgacatttatttcttctggtttgaattgctgcacgtcaagaatcactcgaaatttgtctttgtcatt
tctcattatggaccaaccacaatcttcctctcgttcccaatctatccatggtcgagaaaaattataaggaaat
gatctgaatggtatcgaaggaaaaagttgatctgctcgcaatcctcttccaaaatgttgatccaataatcgat
gtggttgttccaatgcttcccaccaatgggaaaacaatcttggaattaatgtcattcctctcctcatTTTGAA
TTTCTACTTTTCTTCACACTTTCTTGAAAGAATTAACTTTGTTATCAATGCGGAAATACGTGCGCACTCGTCA
CTCGATCTCTGTATACTGACAGCGTATGCCGACAGCGCAACTTTATATATACTCAATGAGGGGGTCTTTAGAC
GCGCGTGAAATGACGACTGAAATTTTCGTGAAAATTCTAGAATATTTTTTGGATCTTTCTAGAAATTTCTAAA
TCGATCAATGACGTAACGTCATATCTATATGTCATGATTATTTATAGTTACAAATTAATAAAAAAAAAGCAAA
ATATTTTCGAGCGAGAATTAAATTTTTTCTATTTGTTATTTCTTTATCGTTAATATTTCCTTGAAAAAATTTA
AAAGAAAGATAAAAATAAATATATTTAAAAAAAAAAAAAACTTTAGAATGTTATTATACGATTTAAAATTATTT
AGAATACATTATTAGAATAGAAGAAATGTACGAAGTCATATTGCAATAAATTTTTCATAAAAGTCTGAAATTA
ATTCATGATTCACACTTGTATATGAATTTACTTTTTCAGATTTTATGCTTATTAACAAAAGTTTCGTCATTTT
ATTTTGTATAGAATCACATCACCTTTACCTCTCCATATAAATTGCATCATTGTCTGCTTAAAGCCTAACAATA
TTGCATCAGCTTTATAGTAGATAGATATACAAGTTTAGTTTCTATATATATGTAAGACATTTATTTTTGTCAT
ATCTAAAAATAAAAATATAAATTAACTATTTAATTAACGATATTTACATATTTGTTATATAATGTTTATATAA
AATATATATTGTCATGTATTGTTATGTACTGTCATATATTGTGTAGAATTATTTAATAAAATCTTATTAAACA
AAAAAGATAGATAAATCATTAATTTGTTAATTTCCTTATTTTGTATGGTTTTCATATAATAATTTTATAATTT
TTAAGAGCTGTGAGATATAATATTAAACATTATTATAATAAATATGTTTTCTTTTTCTTATGTTATCTCATTA
ATTTTTTTTTAAATATTAAATAGTATTTTTTTCCCATTCAAATTTAGTATTTTAGTAACGCATAATAATGTTA

TCATGTACTGGTTTGTATCGATCTTTTCAAACAAGTTCTCGAAGGAAACCGTGACCGTGCGCAATGTTACATA
ACATTTCTATATGCATATGCATTTGCATAGATATGTATAAATATGTCTGTATATAAACACGATCGTTGAACTT
TATTCCTCAATATTAATTTATTTATGTATTTTCAAATATGAATAAATTAAAATCAAAAAAAGATAATTTTTTT
TTTAACTGTTTAGTCGTAACTATTATTCAAATTATTTTTATTAAACTAAGGAGATAGAAAATATTTTAAAATG
CTTAAGATAAATAATTTATACATGTATTTATGTATGTATCCATAAGAAATTGATGTACTATTATTTTTAAGCA
TATATGAGATAATAATTCTCAAAAAGTTGAAAGAGTTTAAAATAATTTCTGATAATTACATTTGATATATTTA
ATAATATATGATTCAAAATATAGAATATATATTTTTCGAAATTTAAAATAAAAACGATTAAAGTTGTCACAAT
AAATTGCATATTTTTTACTCGATTATCATCGAGCAAATAATACAAACAATATATATAAAAATAAAATTATCAT
AGATTAACATCTCATTATGAGAGATGCGATATATTATTGAAAATTATTGTTTTTAATTAAAAAGTTTTTATAA
TCACGTGTATATTACTGGTATTTCATTTTACATCTAGCTTTTGTTTATTAATTATAATAAATCGCATCACATC
ATGCAAAGTTAGAATTTTCTAAATGCATATTTAATAATATATAAATAATATATTATAATAAAAATATATTTAA
CTCTTTAATACTAAAATCAATACATTAACATTATAATTTTAATAAATATAAAAATTTTTATTTAAAATATAAA
AATCAGATATTAAATTTTTATAATCAATAAAAATATGTTATTTAAATGACATATTTTATTATAATATCTTTTA
TAATGTATATAATGTAGAAAATTCTAGAAATTCTTATAGTGATATAAAATAAATATAAAATTATTGATTTCTC
ATGTTTTTGATTCATAAGTGATTGATACAAGTACACGTATTTGCATCATCATTGCATTATCATTGAATAACGT
TCTACATACGTAAGAATGATTCACTTACCATTCATCAAATTTATAATGGACTACATAAGGTAATATATTTCTC
GAATTTCTGGAACTTACCAGAAAATTCCAATAGTTTCATATTTGACAAGTTATAAATTATAAGAGCTTTAATG
AAATAATTCAATCTTAATAAAATAAATGTAAAATAATATATCCTATTAAATGATTTTTTTATTTTAAATACAA
ATATATATATAAAACTTTTATATAATAATAAAATTTTCCACAAAATTAATTATAAATTATGATACTAAATTTT
ATTTTCTAAAATATATATATTTCTTTATATTCACGATAAAGAAAAATAAGATCAATGACCATCTTTTCACTTA
CGTATTTGATACGTAATTATTGATACGTTTATTATATTATCTTAATGTTATTAATTTCAATAATAAAATAATT
TGAAGTATAATAAATATAATTTTTTGAACAGTGCAATGTTAAAATTCTCGTAATACGTGAAAGAAATGTAAAC
AAAAGTAATACGATTCGATCAATCGTATCTAAATTCGTAAATATTACAACATCGTATCTAAATTCGTAAGTAT
TACAACAAAATGACACAAATATCGATCATCATTTGATCGGCTCAAAAATGATAAAACAAATTATGAGAATTGC
ATTTCTGTTCTTAGCCTAGTAACTTCTCGAAACTCCTGCGTATAGACGACGCACCACTACAACGATACTCAGC
CATACATAAATAGCGGACGCTGAGACA**GAACTCCAGTTGAACTTCAAACATTGGACACATCGGTGAGAAGAGA**
TACGCTGCATCCATTTCTTCAAGAGTATAAACACGTGAATTTCGATTACAAATTTTTCAACTATTAAAAGAGA
ATATCGATCGAGGATCTTGAATTCTGACTGAACACACatgatgtcattgttgccgctgttattttctgcctgg
tgggcagatttggatcgtccacatcgtatctgggatcaaaattttggcatgggcctgtatcctgaacaattaa
tctttcccagttcgatcgattcgagaatatattcgccgttaaacaacagagctatgctggatttctactatcg
gccattgtccgagtttttgcgacgtgacgggggtggcacgtcgaccattacagcggacaaagacacgtttaaa 724367
gtgatactcgacatccaacaatttaaaccggaggagatcaacgtgaaactgatcaatcgattggtcgtggtag
aggcgaaacacgaggaaaagaaagacgagcacggtttgatctcgagacagtttataagaaaatacttgttacc
cgaacaagtggatgaagagaaattgacatcgagtgtgtcctccgatggcatattaataatcacggccccattg
aagcagattgaagaaaacttgaacgagaggaacatcaaagtcgagttcacggggaaaccagctcttcatgctg
attcgaaagagcaaacagctgacgagaagaaaccagtctctgagaatgaaaatcaggagctgatagaaaatcc
agagaaaaaataaaATTAAATTTTTTTTATCCTTATAATTTTCCTCTAATTTATTTCTATGTTAATATGTATA
GACTGTTTTTTATTATAATTAATGATGATTGAATGATAATTACGTTATGAACTTTTGTTATAAAATATTGTTC
ATTTTTTTCATAATTTTTTAATAAAAAAAAAACTTTAATAATATTCTATTGTGTAATTTCTACTAATTCCTTT
ATTTTCTATAATAAACTATTAAAAACTAATTTTTGGATAATTTTATCTGATATTTGTTAATTCTTTTTAAAGA
TTTATTCTATTTCGTTCTAAAAATTTTTTTTTGCGAATTTTTAAATTACATATTTGTTGTTAAAATAAGTAAA
AAAGATATAAAATATTAAGATTTTTAAAATTTATTTCTTGTGGACGAATAAATAATTATAATTGTGAAATATT
TTCCATTAAAAATACGTATTATTACGAAACAAGATGGCTCTTTGTGAAAATATAAAAATATTTTACACGATTT
CATTGTATACCTATCCTCTTTAACAATAGAATATCCATATATGGCAGATAGATTACATCTCATTTTGCCATTA
AACAAGATGCAAGAGGATGAGAAACTGGAAACTTACATAGTACAATAAGGGTATTATATTTATTCTTCTTCTT
ATAGCTTTTTTTTATTATTTCATTATTTTTTTATTATTACATTTAATTCATTTCAATATTCTAAACAAATAAT
ATCTTAATTGTCTTTTATACAATACAAATTCTAATAATTTTAAAGATTATTAAAGATTTTAAAAATAGGCGCA
GTATGATCAGAAAAATTTATAATTAACTTGTTAATATAACATATTACAATATAAGACATTTCGATACATGAGC
ATATTTTAATTATTTCGCGCCAAAATTTAGGAGGCAATCTTGCATGTGTTGTGCATTACGCGCAAACTCGTAA
GTAAGATCATACTGCGGACAGAACGCCAATACTTGCATTAAACCTTATATGTATATTGAATCTCAGTGATGTA
TATATATATCACGCATATTGATTATTAATAAACCGCATTTCTAGGGTTTTATTTGAAAATATCATTAAAATCC
TTTGAATTGCAAGTTTTGTATAATTCAATTTACATATAAATATAGTTTTTTTTAAATAAAATAATATTATTAG
TATAAAAAATAAAAGTTATTAATTGATAGTGTTGTAATATAAAAAATTTAAAAATTATTATTACTTGATAGAT
TTATATTATATCAAAATATAAAATATATTATCTAAATTTGAATATTAAGCTATAAGATCATTCTTCTTTTACA

AACTACGAGAAATTTGTTAAACATTTTTATAATAATATATTTTCGATTTCAATTTATTAAATTTTATTTATAA
ATTATAAATCATAAGAATTTCATAAATTTCATTGATCAAATATTCAATGATAATGAAATAAAACATTAGTTAA
AATAATGATAAATAATATTTAAGTTATATACTTAATAATTTATTTAGTTTTATTATTTTCCAAAAATTTCGCA
TTGCGCCTGCTTGTAAAATTTTTAGGTGCTGATTCGTTCGATTGAAGCGCCTTCCTTTATATGCT**CGGTCATG**
TCGAGAAATACAGCAGTCCGTGTGGAACAGTGAATCTATCACAAACTAATCGATTATTTTAAATCAAAGAGCC 724405
AAAGTTCAACATCAAGTTCTAGTCTACGTAGCTCGAATTAGTTTCGCAAATCTAAAatgaatctggaaagttt
gcttcttccttacttatgggagaatatggaacgtgctcattccactataaatccgaatcttagagttagaatc
aatccagagaatctgggctcgaaaatcatgcacgtgtacgatttcctaccgcaaaaaacgagtccaaaattgc
acatggactattacagaccatggggtgaattgttgcgcaaaagcgaaggtggtgcatcaacggtgacagctga
caaaagccagttccgtgtggatctggatgtccaacaattctcacccgaggaaatcaatgttaaagttgtcgac
cgtttcgtgattgtcgaagcaaagcatgaagagaaagaggacgagcatggctggatttccaggcaattcatga
ggaaatacataattcctgaacaatgcgacatcgatcaagcgtcctcgaaactttcatctgacggtgtcctttc
gattattgtaccacgcaagcaaaaagtcatttcggaaggcgaaagggtaattaacattgaacatactgggaaa
ccgtgcgatgcgcaaaatgaggaaagaaaagagaaagaggaggacgtggagtaaTAACTCGAAGTCTCAATAC
GGAATAATCCAGTGATTTTTATCACCTTTACTGTTTCCAGCTCTTACCGGAAATATTTATTTAGTGTTATATG
AAAAAAATTAAACATATTTACGTATTAAATACGTCTTAAACGTATTTAGATGTGTATAGCTATTATTCGTTGC
GCGAATCTTCTTGTACGTGTAAAGTACAAAATTTGTTGAATACATATGATATTATCCATTATTTTAAATAATT
TTATTTTTATATATGAATAAGTATATTAAATTTATTTCTATTCGTCATTTTGTAATATTTTGTATTGTCTTCG
AATATTTTTTTATTTTATTTTATTTATATCCTTTTAATATGTTTCTATCTCTTTAATGATGTGATGGCAAATG
ATGATATTGATCCTGTGTACTATGTATGAAAGTTGTTACTGTATGTGTAAAGTTTTATAAGCGGTGGATAATA
TATCGAAATTTATTGTAATTGTAAAACAAATCTAAATGATCGTGAAGAAAGTAAAGAAAGTAGATTTTATTTT
TATTGCATTTATTTGATAAATGCAAGTTTGTTTTTAACATGTGAAAATTATAATTAGTTCAAATTATCAAAT
TTAAAAAATACTTATAGATGTATGTTATCATTAAGAAAATATTAACTCTAATAGACGTAATTTTAGATGTAAT
TATTATTTTAGATGTATTTATTAAATAATAAATTACATATTCATATTAATTAATATTATATATATATATTTTC
ATGCTTCATAAGAAGCATTTGCATGTAAATAGATGTAATCAATAAGATTAGATTTAATGATAATATTATTAAT
GATTATATTAATTAATGATTTAATTATTGCAAGAATTATTTCAAGAAAATATTGTATTTTATTTTATCTTTCT
CTATTATTAAATATATTGGAATTAAATCAAATAATTATATCAAATAAATAATATTACATTGTTTTATTTTTAA
TATGTTGTTTAATCATTAAAATTATTTTGTAAATATCAATTTTATAATTTTTCTTCACATTTATAAATGTAAA
TGATGTTCTATATAATTTAAAATAAATTTAAAAACTATTTATTTATATTATAACAAATTTTCACATAATCCTC
TATTATATTTAAAATTTTTTTATTTCTCTTATTGTTTACCATATGAATTATATAATTTTCTTTTTCAGGAAAA
AACGTCATATAAACGGAAATATGCAGATATCAAAATAATTTATTAAATTCAAAATTTAATAACCTTTAAATGA
AAAATTTTAAGAAATCAATATATATCCAAAATGCTTTTATAAAATATTATTAATTTTTATTTATTAAATTTAT
TATTAAATGTTATATAATTAAAGAATTATAGAATTTTATTAATAAAAAATAAATGTAATTAAATTTTTTATCT
TTTCTTTTATCATTTTAAAGATATGATTTATCTGAAAAGTAAAAATATACTGTTACTTTTAAGCTTATGATAC
AGCATATTAAACATATCACATTTATGATTCATAACAAAATATTGTGCAATATATCATATTATATACAATCATA
TCATGTCAAAAAATTTTATAAAATATCATAATTTATTTCTATCAGAAGAGTCGATTTGACATCTAAAAACATT
CTTAAGCGTCTAAAGACATTATTACGTTAAAGAACTTGTTCTTATATTAGAATAGGTATACATAGTAACACGG
TTTTCATAGACAGTGGCGTTAAACATAAAACGTACATGCTATGGTTAAGTTACGTTATATTGCGCAAGTTGCT
ATACAAAACTGATCTAAGATACGAAAACGACGATTGTATTCAAGGGCAACTCGACATTTTCCAGGGTCTTCTC 724449
GAATGCGCGCGGTGGTTCTATCCCCACCATTTTCCTTAGTGCGCAACAAATATGTATAAAAGAGAACTA**CCTA**
GTTGCCGACAGTTGATGTCGACGTATGAACGATATAACAGTACTACGATTAATCAGCTGCAAATAAATCAGTA
ATTTATACGTTTCATAAAATTAATAACAAAGGTAAAGCATTTTAATAATTTGTTAGTGAAGCATTTAAATTAA
TTATATTCGGAAAGTGGGGAAACATTCCTAACAACATAAACTACAGTTTGAATTGGATATTGAAAATACTTGC
ACAatgtcattaataccaatgatgttttccgactggtgggaagatctagaccgacctcatcgtctttgggatc
aacattttggcacagcaatagatctagatgattttaatgatctagattcacttggttcagaagttctgctata
tcgaccacataaacgcggcagaagacatcatcgtcataatcatcatccattcttgaaggctttcaacaaaagg
catggtcgcggtgcatctattgttcaagctgataaagacaaatttcaagtgacattggatgtatcacagtttg
caccggaagaaattactgttaaagtagtagatcagaaagttgtcatcgaagctaaacacgaagaaaaaggga
tgaacatggttgggtatccagacaatttgtcagaaaatatatcgtaccgtcacaatgtgatatcaatcaagta
gaatcacatttgtcttccgacggtatcttatctattacggcaccaagaaaagaacctttacaatctagatcaa
atgagagaacagtgaaggtacattatacaggtgaaccagctttgactaattttgacgattcttcaaatgacgt
ttctgaatcacaaagagaacaacaaattccacaacgagaacaatcacaatcacaatcacagcaaaatcaacaa
ttacaaaaccaacaacatcaacaacatcagcgttgtaaaaagggcagtaaaggtgtataaGTCATGTTCATTA
TTTTTTTATTTTCATCTTAATTAATTTTAATTTTTTCCACCACAATAAACATAATTTATAGTATTATAAAACA

TTATATAAAACTTATATGTATATTAAACTTTACATGCACAAGTACAAAATTGAAGACAAATATTGTATTAATT
ATTTCTTTAATAAAGTAATAAAGAAACTTTATTTGATTTAAACTTTTGTTTGTTTATTTTCTTCCTTTCCATG
ATAATATTCCACAAGATGATAATAAAATTCAATCAATTATTCAAAAATTGTTATCTTCTTTTCCCCTTTCCAA
TTTTTAGACATGCATATAATAGATAATATATTTTAAAACAATTTTAAAAAATTGTTAAAAAAAAATAATTCTT
ATTTATTTGCTCTTTTTCTTTCCATTATTATTAAATATCTATCACAAGACAATTTTAAAAAATTATTCTAAAA
ATATTCAAACATATTTTAATTGATAAAAAAAATTATTTTCTCATAAGTTATTTTCTATTTTCAAATATATTAT
ATTTTCAAATATAAAATATCTTAATTTTTCTTATATCTTAATAAATAAATTGATGTAATACATATATATTTAT
GTATATGTATTGAAATGATATTTTTTTATTTAATAAAGAATAAATATTTTTATGCGAAATTAATAAATTGAAT
CTTTTATTAGAGGTTGAGATTTCTCATTTAGTTAACAATACTTGTTAACTGTTAAAAATAATTATATATTTTA
TCTTATAACAGAAACATCCAGAAACATCCAGAAACATCCAGAAACATCCAGAATTTTTTAGCGAACGCTGACC
TGATTAATGATAATCATATTTACGTGTACTTATGTGAGCTTGCATCCATATATGTACAGTTAATCATAAAAAT
ATTTAAGAGTATACATTTGTTTTTAAATGTATACATTTTATACTATAAATACATTTTATAATAGAGAAAAAAA
TAATTAAAATTTATAATATTTTCAACAAATGTTTATTATTTTGCACTATAATAATAATTTAATTCAATGTTTG
TTGAAAAACGTATTATGATACATTGAAAATTTTGTGACAAATTTATTAAACATGAAAATATTTTATATTTATT
AATTTATATAAATAATATGAAGAAATTTGATTCAAAAATAAATTGATCAATGCAATTTGAAAAAATTATTATT
ATATTTTAAATAATTATTAATATATATTCATCATAATAATTATGTGGAAAAAATATATAATTTTTTTATTTAT
TAAGTTTATTAATTAATTTGATTAATTAATTTAAATATATTACTTAAAAAATTATATAATATTGTTCTAATTA
TTTATATATAGAAAAATATATATATAAAAATTGTATGTTAAAAAGTAATACGAAATACATATGCAAATGATCA
GATATTTTTGTGACGAACTGTACGTATAAGATGGTAGCTTAGAAGCACCGAGAAGGATCAATTTTATACTGTG
CAAGAATAGATTGCAAACACAGTGAATAAAACTAGCCTAAAGATCGATAGTACACATATTCATACAAATTCAC
ATAAGTATTTTAAATAAGACTAAAATTCTAATAGATTTTTATAATGTATTTAAAATAATAGAGCTTAGTTTAA
AAAAATCAATATTATTTCAATATTATTTTTATTTAAAAAATATAAAACAAAAAATAGATGTTATTAATAATAA
GTATGAGTTAGTTAGATTTTAATAGAATATGTTGAAAATATTATTCGTGTAATAATCTGTTGGCTGAAGTTAT
TCATACAGATTTTATATGTAGGTATATTTATATCAAGATCATATATTATCACGAATATTTTGAAATTTAAAAG
TTTTTAATCTAAATTGAAAATAAGATTAATTTAATAATTTCTATATTTTCAACATTAATGTTGAAAATTATAA
AAATATAAATTTTTATGGAAAAAATGCGTTTAATATGTTTTTAATATAGATATTATTTTCTATGATATTCGCT
AAAATTTATATGTTAGTATATAGTAATATTGTATACTTATATATATTACATATATAAAATATGATATTTTTTA
ATATTTTTTTCATTATATTTATAATACATTTAAGTTAAAACATTTTGTAGACATATCGAGAATTTCATGATAA
AAATGCATCAAATCATTGATATTATATCAAGAGATAAATCTAAAAAAATAAGGAATTTTTTATAAAATAATTG
TCTTGCTAGAAAAAAAATTACTGGAAAAAATTAATATCATAAAATATTTAAATATTCTGCGAGATAAAATATT
CTAGAAATAAAAGATAGAAATAATAAATGAAAAGAATGACATTCGATGTTTTGTATAATATATATTTTAATAT
GCCGGGTGTTTTACGATTGCACAGCAAAGGATTCCTTTTAATTGTTAAATTGTTAAATTTGTTTGTTATATGT
ATAATTATTGCATATAATTCAATACTTCAAGTCTCTATAGACAATATAAATATCTAAAAAGATCTTTCTAGAT
AAATAAAATATAGTATTTTCTCGAAAATGTTCGCAAATAATAGAATGTTCTGGACACTCCACCTATCGGTAGC 724448
CGGTGCTATAAATACGTGCGCCGCGATCAGTAATAGCAGTATACAGAAAACACTCGAGAGAGAAGCTTGACAA
GCTTATTGTAAACATTAAAAGTGAACATTTCGAAAAAGTCTGTGACTTAAGACATTACTATATCAAAAATCAA
ATTCAGCTAAA**ATGTCTCTGATTCCATTGCTGTTCTCTGATTGGTGGGAAGATTTGGATCGTCCTCATCGACT**
ACTTGATCAAAATTTTGGTTTGGGATTATATCCCGAGCAATTATTGAATTCAAACATTCTTGATCAATATATC
TTACCCAATCGTAACCAAAGATTAAGAAATCCATTGATATATTATAGACCTTGGGGTGAACTTCTGCGAAAAA
ATGAAGGAGGAGGCACATCAACTGTGAAAGCGGATAAAGATAAGTTCCAAGTAATTTTGGATGTTCAGCAATT
TAAACCAGATGAAATTAATGTCAAAATCGTCGACAAGTGTGTTGTCGTCGAGGGGAAACATGAAGAAAAACAA
GATGAGCATGGTTGGATTTCGAGACAGTTCACCAGAAGATATATGATTCCTGAGCAATGCGATATTGATCAAG
TAACATCTAGTTTATCATCGGATGGTGTATTGAACATTACTGCACCTAGGAAGGAGCAACCAAAGATCCAGAA
CGAAAGAAATATCACCATCGAGCAGACGGGTAAACCGGCATTGAAGGAAAACACGGAGGAGAAGAAAGAAGAG
AAGAAAGAAGAAAATTAGAATCAGAAAACATGTTGAATTATTGATAATATTGTGTTCCTGCTTCTTACTGCTT
TCATATTTCTAAAGTTGTCTATTTTGTCATTTATTTAATTCGATTTTTGTAATATAGATTAAAATTTTTTTTA
AAATAAATATTCGATAAAATATAATCTTGAGTTTTTTTTTTTTATGTCCTTTTTTCATTTAAAATATCCTTCAG
TTATTTTAATATATCGAATGTCTTCTTTAGATTATGCAATGTTAAATTTATGGATATTCATATTTCAACATAC
TATTTTTAGAATAATGTGTATATATATATGCATATAATTTGAGAGAATTCAGGATTTCATTATTTTAGGATAA
TAAAAAATAAATAATAATAATCAATTGAAACTAATAACTATCTAAAAAATAAATATTACTTTTTGTTCATAAT
ATATTATAAATTTTTCAAAATCATTTTAATAAATAATTTACAAAATGTAATAATTTCTTTATATATTTGAAAT
ATTTTCATACAAATCTATATAATAAAAATAGATTTGTAAAAGATTATATATATATTTGCAATTCTTATTAAAA
ATTTTAAGCAATGTAACTCAAAAATGTAATGTATGTAATATTAATTTATAATTTATTATTTATTGCTAGCCCA
CCATTTTGTTTTTTAATACACGACTTTATGTACAATTAATGTAAAACTAATTCAAGCTTAAGTATTTTATCTC

AGAGCATTATATATAATATTTAAAATTACAAATATAAATAAAATCTGTAATTATAGTGGCTATCAAATTTTAA
ATTATATTACATTAAATTTAATATTTTTTGTTTTGTAAAGAACAAATAAAAATATAATATTTAATACGATTTT
TACAAATGACACAAAACATGAAAGAATTTAATACTGCAAATATATCCTATTTAGTATTTCATTCGTTGTAGGA
GTTTTTATCTAAATGCTTCTAATAAAACAGTATAATTACGGATTATCTTGAATTGACAAAACTTTCATTTTAT
ATTAATTATTATAACATATTTTTCCAATGTACAATACTTAATTATTGTCAATAAGAAAAATTGAATTTTTGAA
TCGACGTTAAACGTTTATGTCGCAAGCACCATGAAGTATTACATATAATTTTTACTAATAA

Supplemental Figure 7. Amplification curve (A) and melt curve (B) traces for the LAMP assay for the *724367 l(2)efl* gene.

A

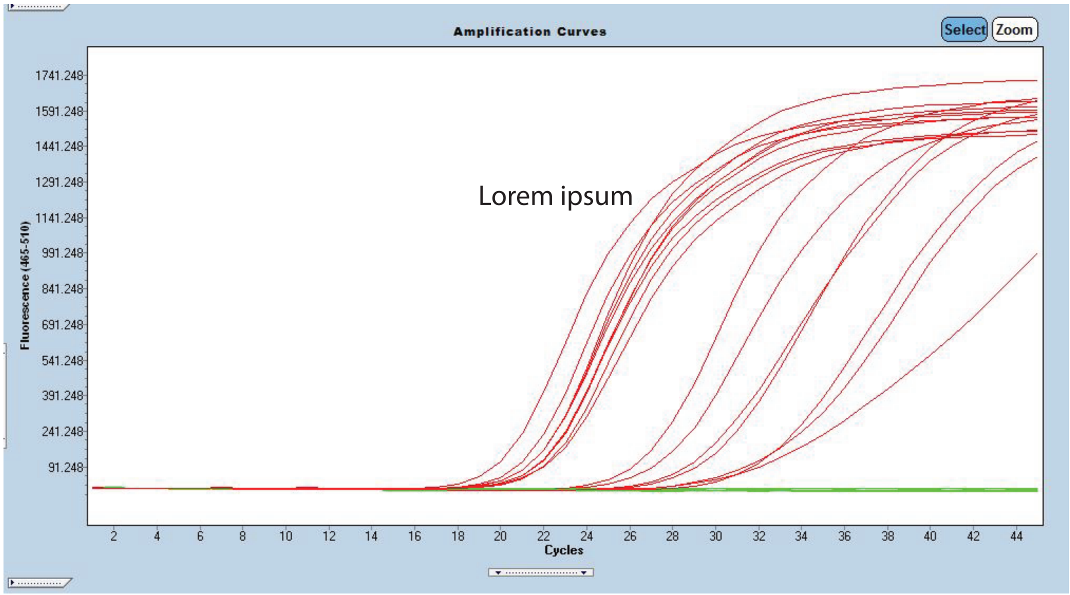

B

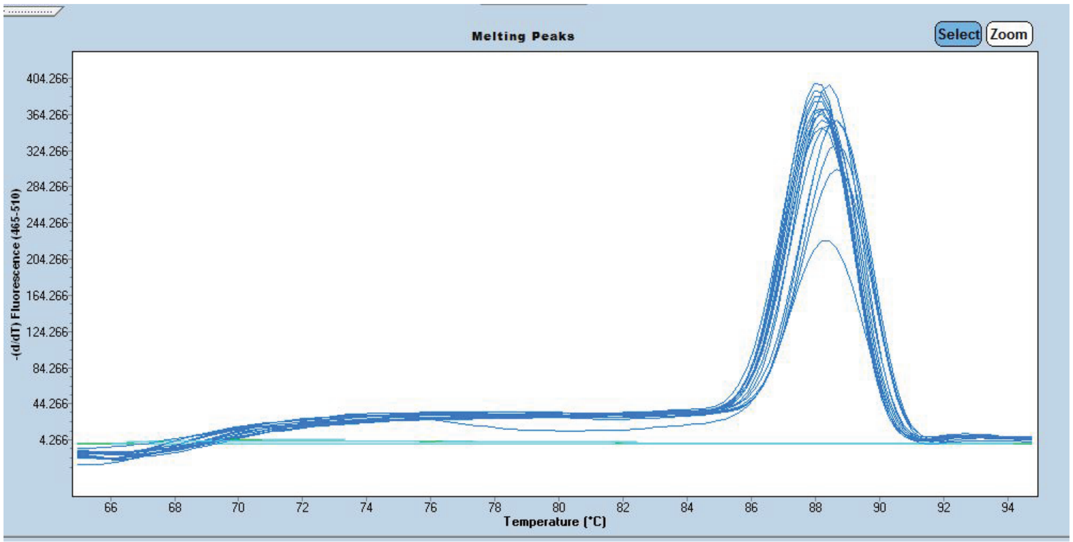

Supplement: Supplementary file 2 — Supplementary Figures. [file 41598_2021_1547_MOESM2_ESM.pdf]
